# Indicators for optical oxygen sensors

**DOI:** 10.1007/s12566-012-0032-y

**Published:** 2012-11-24

**Authors:** Michela Quaranta, Sergey M. Borisov, Ingo Klimant

**Affiliations:** Institute of Analytical Chemistry and Food Chemistry, Graz University of Technology, Graz, Austria

**Keywords:** Bioanalysis, Oxygen indicators, Luminescence, Metal complexes, Quenching

## Abstract

Continuous monitoring of oxygen concentration is of great importance in many different areas of research which range from medical applications to food packaging. In the last three decades, significant progress has been made in the field of optical sensing technology and this review will highlight the one inherent to the development of oxygen indicators. The first section outlines the bioanalytical fields in which optical oxygen sensors have been applied. The second section gives the reader a comprehensive summary of the existing oxygen indicators with a critical highlight on their photophysical and sensing properties. Altogether, this review is meant to give the potential user a guide to select the most suitable oxygen indicator for the particular application of interest.

## Introduction

Oxygen is by far one of the most important chemical species on earth since it is essential for life. Measurements of its concentration are of extreme importance in many different research fields such as: medicine, chemistry, environmental and marine analysis, molecular biotechnology, bioprocess control, food packaging, and industrial production monitoring. In the majority of the cases, it would be ideal to monitor oxygen concentration continuously which implies the use of oxygen sensors: a class of chemical sensors and by definition “*a miniaturized device that can deliver real-time and on-line information on the presence of specific compounds or ions in even complex samples*” [234].

Several methods for oxygen detection exist and can be classified on the basis of the principle used in electrochemical (amperometric, potentiometric, or conductometric), optical (absorption changes or photoluminescence), and chemical (Winkler titration).

Since its development, the Clark electrode has been considered the conventional method for the measurement of oxygen concentration because it is quite robust and reliable. However, in the last three decades, optical sensor technology has received increasing attention due to the fact that optical oxygen sensors can be rather inexpensive, are easy to miniaturize, can be used remotely, are virtually noninvasive or minimally invasive and, most of all, do not suffer from electrical interference nor consume oxygen [3].

Optical chemical sensors can be divided in several subgroups depending on the working principle applied; practically all spectroscopic methods have been used (absorption spectroscopy, reflectometry, luminescence, infrared and Raman spectroscopies, interferometry, and surface plasmon resonance). The majority of the optical sensors developed for oxygen detection rely on quenching of the luminescence of an indicator dye by molecular oxygen [153].

Typical layouts consist of a luminescent dye, whose optical properties are reversibly influenced by the presence of molecular oxygen, which is usually incorporated into a polymeric matrix and deposited on a solid support (planar waveguide, microtitre plate, or optical fiber). Nano- and microparticle-based oxygen probes have also proved to be important analytical tools.

The field of application plays an important role in the choice of the indicator dye and, as a consequence, of the matrix material and detection method. For example, when measuring oxygen in live cells or in tissues, it is necessary to take into account the autofluorescence generated by the presence of biological substances such as proteins, DNA, and melanin. In such cases, in order to minimize absorption and scattering of the excitation and emission light in the tissue, it is preferable to employ indicators that show longwave-shifted absorption (590–650 nm) and emission (730–900 nm) bands [63, 223]. On the other hand, when measuring ultrafast oxygen dynamics, for example in breath monitoring application [33], it is crucial to use optodes with a very fast response time which can be achieved by employing very thin sensing layers and indicator dyes possessing exceptional brightness.

The scope of this review is not only to provide the reader with a selection of recently developed oxygen indicators but also to give a feeling about the area of applicability with a special focus on bioanalysis.

## Photoluminescence and oxygen quenching schemes

Photoluminescence is the emission of photons produced in certain molecules during de-excitation and is one of the possible physical effects resulting from the interaction between light and matter.

**Fig. 1:**
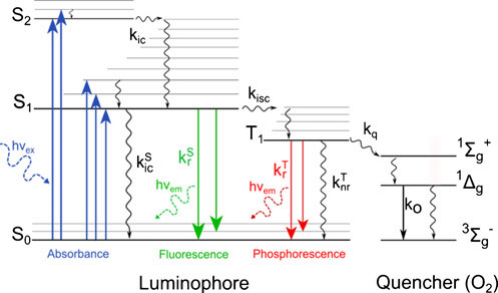
Perrin–Jablonski diagram

When a luminescent molecule absorbs a photon, it is excited from its ground state (*S*_0_) to some higher vibrational level of either the first or second electronic state (*S*_1_ or *S*_2_). This transition occurs in about 10^−15^ s and the subsequent possible de-excitation processes can be visualized by the Perrin–Jablonski diagram in Fig. 1.

The first process to occur, after absorption, is the internal conversion from the excited vibrational level to the lowest vibrational level of *S*_1_, which takes typically 10^−12^ s or less. Internal conversion is a nonradiative transition and it is generally complete prior emission. From *S*_1_, two major radiative de-excitation processes are possible; the first one is fluorescence emission which is a spin-allowed transition (it happens with no changes in multiplicity) that has a high probability of occurrence; fluorescence lifetimes are typically near 10^−8^ s. The second possible process is phosphorescence emission from the lowest excited triplet state *T*_1_ which requires nonradiative intersystem crossing between two isoenergetic vibrational levels belonging to electronic states of different multiplicities (*S*_1_ → *T*_1_) and therefore has a lower probability of occurrence. Transition from *T*_1_ to the singlet ground state is forbidden; therefore, the rate constants for triplet emission are several orders of magnitude smaller than those of fluorescence and the lifetimes usually vary from 10^−6^ to several seconds. The presence of heavy atom within the indicator, either as the central atom or as a substituent in the ligand, usually greatly increases the probability of intersystem crossing (up to unity). This result can promote highly efficient phosphorescence and shortens the phosphorescent decay time.

When an excited molecule returns to the ground state, it emits light at a longer wavelength and lower energy compared to the absorbed light (Stokes’ shift); this phenomenon is more pronounced for phosphorescence emission since it occurs from a lower energy state.

In the presence of molecular oxygen, the photoluminescence of such molecules is quenched via a radiationless deactivation process which involves molecular interaction between the quencher and the luminophore (collisional quenching) and it is therefore diffusion limited. The mechanism by which oxygen quenches luminescence is not yet completely clear, one of the proposed mechanisms suggests that the paramagnetic oxygen causes the luminophore to undergo intersystem crossing to the triplet state while molecular oxygen goes to the excited state (either ^1^Δ_*g*_, the first excited state or ^1^Σ_*g*_^+^, the second excited state) and then returns to ground state (^3^Σ_*g*_^−^, the triplet state; Fig. 1) [124]. The formation of singlet oxygen (^1^O_2_) is a direct evidence of the energy transfer mechanism and often the quantum yield of singlet oxygen formation approaches unity. However, quenching mechanism can also occur via electron transfer which was demonstrated to be rather efficient for example in the case of iridium cyclometallated complexes [56].

Independent from the predominant mechanism (energy or electron transfer), the kinetics of collisional quenching by oxygen is very well described by the Stern–Volmer equation (Eq. 1)

1 $$ \frac{{{I_0}}}{I}=\frac{{{\tau_0}}}{\tau }=1+{k_q}{\tau_{0\ }}p{O_2}=1+{K_{sv }}p{O_2} $$

Where *I* and *I*_0_ are the luminescence intensities in the presence and absence of the quencher, *τ* and *τ*_0_ are the lifetimes of the luminophore in the presence and absence of the quencher, *k*_*q*_ is the bimolecular quenching constant, and *K*_sv_ is the Stern–Volmer quenching constant.

It is often the case that in microheterogeneous environment (e.g., in polymers) Eq. 1 does not adequately describe the quenching mechanism; in such cases, it is preferable to use a second equation from the so-called two-site model [35] (Eq. 2)

2 $$ \frac{I}{{{I_0}}}=\frac{f}{{1+\mathrm{K}_{\mathrm{sv}}^1\mathrm{p}{{\mathrm{O}}_2}}}+\frac{1-f }{{1+\mathrm{K}_{\mathrm{sv}}^2\mathrm{p}{{\mathrm{O}}_2}}} $$

where *f* represents the fraction of the total emission for the first site and *K*_sv_^1^ and *K*_sv_^2^ are the Stern–Volmer quenching constants for the two sites. The two-site model assumes the existence of two different environments with substantially different accessibility for oxygen. Although this model is physically meaningful only for luminescence intensities, in most cases it can also be used to fit decay times dependences.

Apart from the two-site model, other models exist that are able to describe the degree of heterogeneity of a sensor; for example the log-Gaussian distribution in *τ*_0_ and *k*_*q*_ [161]. The model assumes that the heterogeneity of an optical oxygen sensor is manifested as a log-Gaussian distribution in rate and therefore is controlled by two kinetic processes: the intramolecular deactivation of the excited state of the luminophore (1/*τ*_0_) and the intermolecular quenching of the excited state by oxygen (*k*_q_·*p*O_2_). Such model is able to generate model parameter values which are physically plausible and consistent at all partial pressures of oxygen.

## Sensing methodologies

Although some of the oxygen sensors in use rely on absorption-based measurements, the majority make use of the luminescence quenching of an indicator dye. Luminescence sensing requires a change in the spectral properties of the indicator in the presence of oxygen. Changes can occur in the form of emission spectrum, luminescence intensity, anisotropy, or lifetime of the sensing probe [124].

The most direct sensing method entails the measurement of changes in luminescence intensity in response to the presence of an analyte. Unfortunately, it is often inconvenient to rely on intensity changes since they can be influenced by a wide variety of factors (e.g., luminophore concentration, photobleaching, light source intensity, scattering, coloration of the probe, etc.) and therefore alter the results of a measurement. Hence, it is important to use alternative methodologies that are independent on these factors, such as ratiometric or lifetime-based measurements. Usually, the choice depends on the cost of the equipment, the availability of indicator dyes, and the field of application.

Ratiometric methods are often preferable for imaging application and typically the sensors contain both an oxygen-sensitive indicator and a reference dye usually incorporated in the same matrix. The latter has to be unquenchable by oxygen and photostable in its presence; the emission spectrum of the reference dye should have little or no overlap with the one of the indicator dye and there should be no energy transfer between the two dyes. Oxygen concentration can then be determined from the ratio of the luminescence intensities of the indicator and the reference dye measured at two different emission wavelengths. This is usually done by using bandpass filters or, alternatively, by attributing the emissions of the components to different color channels of an RGB camera [128, 212, 228]. A more elegant solution is an application of tailor-made indicators which possess dual emission (oxygen-sensitive phosphorescence and oxygen-insensitive fluorescence) but very few examples have been reported so far [244, 254, 256, 258].

Ratiometric oxygen sensors can be applied, for example, for in vitro measurements to image O_2_ concentration in cells [43] or for noninvasive real-time monitoring of oxygen levels in live cancer cells under normal and hypoxic conditions [135]. It should be mentioned here that ratiometric imaging is still affected by light scattering because this effect is wavelength dependent.

An alternative property that can be used to monitor oxygen concentration is luminescence lifetime (τ). The lifetime is the average amount of time a luminophore remains in the excited state following excitation and it can be measured either in time domain or in frequency domain [124]. In time domain, the sample is excited with a pulse of light and then the time-dependent intensity is measured; the decay time is calculated from the slope of the a plot of log *I*(t) versus *t* (slope = −1/*τ*). A short delay between the excitation pulse and the measurement allows the complete elimination of short-lived background fluorescence.

In the frequency domain method, the sample is excited with intensity-modulated light. The emission of a luminophore is delayed in time relative to the excitation; the delay is measured as a phase shift (*φ*) and can be used to calculate the decay time:

3 $$ \tau ={{{\tan \varphi }} \left/ {{2\pi f}} \right.} $$

where *f* is the modulation frequency.

A two-frequency phase modulation technique can be very practical when measuring intracellular oxygen concentration in plant tissue since it allows discrimination between the luminescent lifetime of the autofluorescence of the plant tissue and the phosphorescence of the indicator dye [198].

Pulse and phase techniques are theoretically equivalent and provide the same kind of information; each has its own advantages and drawbacks. As mentioned earlier, lifetime measurements overcome the disadvantages of the intensity-based measurements. Luminescence lifetime, for example, is an ideal parameter to measure in biological system where the exact concentration of dye after cellular uptake is difficult to determine and replicate accurately [171].

Imaging of oxygen distribution has become a popular technique in different fields of application such as biomedicine [158, 257], marine microbiology [75, 86], and biological systems (intra- and extracellular oxygen distribution) [57, 130].

Luminescence imaging can be performed either through intensity- or lifetime-based measurements. One of the first works on intensity-based phosphorescence imaging was published in 1988 and used to measure oxygen distribution in perfused tissue [192]. Lifetime imaging techniques were developed for the first time two decades ago, and both time-domain (pulsed) [229] and frequency-domain (phase-resolved) [125] have been described; in 1997, the first noninvasive technique to measure oxygen concentration on nonplanar surfaces (e.g., human skin) was described [92].

Lifetime imaging has two major advantages over intensity-based imaging: it consents the enhancement of contrast and it allows the suppression of background fluorescence; also, lifetime imaging does not depend on intensity variation that can derive, for example, from photobleaching or variable indicator concentrations and calibration-free sensing applications are feasible [96]. A frequently used pulsed technique for the determination of fluorescence lifetime is the time-correlated single-photon counting in which the decay time curve is obtained from the integration of many pulses recorded over time [196]. This technique requires complex instrumentation and is less feasible for phosphorescence lifetime imaging because of much longer acquisition times; therefore, a second methodology called rapid lifetime determination (RLD) became rather popular [95, 96, 238].

**Fig. 2:**
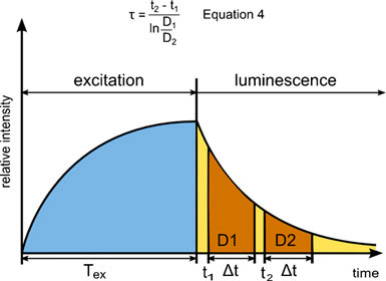
Schematic representation of RDL measurement

The principle for image acquisition in RLD is briefly the following: the measurement starts by switching on the excitation source which illuminates the sensing material, the luminescence intensity increases until equilibrium between absorbed and emitted energy of the indicator dye is reached. Then, the light source is switched off and the shutter of the camera is opened to allow luminescence and ambient light to reach the charge-coupled device (CCD) chip at two different time gates (*t*_1_ and *t*_2_) of identical period (Δ*t*); the lifetime is therefore proportional to the ratio of the integrated photon counts *D*_1_ and *D*_2_ (Eq. 4 in Fig. 2) [139].

In recent years, another technique appeared and became rather popular: multiphoton microscopy, which is based on excitation of the dye by simultaneous absorption of two or more photons. It is usually used with fluorescence dyes to visualize either tissues or cells with high resolution. The advantages of this method rely on the fact that the near-infrared (NIR) light used for excitation penetrates deeply into tissues for which is not harmful, the background fluorescence is absent, the spatial resolution is significantly higher, and the photobleaching is lower than for other techniques such as confocal spectroscopy. As a consequence, some probes using this methodology have been recently developed [74, 131, 195]. Unfortunately, the state-of-the-art luminescence indicators possess very low two-photon absorption cross-sections and that is why rather sophisticated design of new probes is necessary.

## Application of oxygen sensors in bio-analysis

Optical oxygen sensors have been developed for a wide variety of applications and in this section of the review, the ones inherent to bioanalysis will be highlighted and briefly described. We will focus on the following bioanalytical applications: oxygen content measurements in blood (diagnostics), measurements in bioreactors or in cell cultivating flasks, imaging intra or extracellular oxygen distribution, applications in marine biology, and measurements of photosynthetic and respirometric activity [16]. Finally, the role of oxygen as a transducer for enzymatic biosensors (e.g., for glucose sensors) will be discussed.

### Medical diagnostics

Assessing a patient’s need of oxygen is of extreme importance in critical situations such as during surgery, recovery, or while a patient is under intensive care treatment. Sampling of arterial blood and the subsequent analysis suffers from the main disadvantage of not being a continuous method: it is not feasible to sample more than two or three times a minute and oxygen content can fluctuate widely during periods of few seconds.

Back in 1942, Millikan [160] developed the first original oximeter which in concept was very similar to the modern pulse oximeters but suffered from the inability to effectively compensate for the pulsatile variations of the arterial blood. Pulse oximetry was developed by Aoyagi in 1972 [203], who had the idea to measure only the pulsatile changes in light transmission through living tissue to compute the arterial saturation.

The role of a pulse oximeter is to determine the “oxygen saturation” in blood which is defined as the ratio of concentrations between oxyhemoglobin and the total hemoglobin; the method is noninvasive and as a typical intrinsic optical sensor, it does not require any indicator. Light at two different wavelengths is allowed to pass through a part of the patient’s body (usually the fingertip or the earlobe) to be then measured by a photodetector. Today’s oximeters use a pair of light-emitting diodes (LEDs): a red (660 nm) and an infrared (910 or 940 nm) one; at this two wavelengths, hemoglobin and oxyhemoglobin exhibit a very different absorption, therefore, their concentration’s ratio can be calculated from the ratio of the absorptions of the red and infrared lights.

Luminescent oxygen sensors have also been applied in medical diagnostics: for example back in 1994, a portable system for in vitro test blood analysis (OPTI system) was developed and commercialized. The system consists of a disposable cassette containing six different optodes for measurement of pH, oxygen and carbon dioxide partial pressure, sodium, potassium, and calcium, all by fluorescence. Additional cassettes are available for determinations of chloride, glucose, and urea [219].

More recently, additional applications of luminescent oxygen sensor for diagnostics have been emerging such as the control of wound healing processes [200], the measurement of transcutaneous pO_2_ [17], and of tumor hypoxia [258], to mention only some.

### Bioreactor and cell cultivating flasks

Continuous measurements of dissolved oxygen (DO) during cell culture are very important not only to control cellular differentiation, viability, and proliferation [79, 215, 248] but also when designing the scale-up from small-scale culture to mass production [89, 255]. DO measurement is particularly important in shake flasks for cell cultivation since, for example, in such devices the oxygen transfer rate is lower than in stirred bioreactors; therefore, accurate measurements are essential to avoid biological misinterpretation [199].

Luminescence-based oxygen sensors are very attractive for biotechnological application for a number of different features such as the fact that they are noninvasive and generally nontoxic since they can be easily incorporated in matrices that are not only biocompatible but also nonreactive and non-irritant to culture media [110].

There are many biotechnological processes such as the synthesis of penicillin or yeast fermentation, which require a sterile environment; therefore, the optical sensor used to continuously measure the oxygen concentration in those bioreactors needs to be autoclavable and to withstand sterilization (e.g., high temperature and humidity). Voraberger et al. [224] developed such sensor which was used to measure oxygen concentration in a fermenter; the results showed good comparison with the ones obtained with a Clark-type electrode, which has the disadvantage to suffer from inadequate signal stability, slow response time, and electrical interferences.

Optical oxygen sensors were also developed for online measurement of the biocatalytic activity of enzymes in microtiter plates by integrating the sensing layer into the bottom of each plate [80]; such devices are of potential interest for the screening of aerobic cell activities, biological degradation of pollutants, and for toxicity tests.

### Intra and extracellular O_2_ imaging and DO in tissue

The application of optical oxygen sensors in the measurement of oxygen concentration, particularly in living tissue, has the advantage of being noninvasive and it can be used remotely.

Inter- and intracellular measurements of dissolved oxygen can be potentially performed with the use of a ratiometric fiber optic sensor which was developed and characterized by Park et al. [180]. The described sensor exhibited excellent reversibility, minimal photobleaching, and fast response time which are fundamental qualities to measure oxygen in biological samples. On the other hand, the size of the fibers, with tips ranging from 40 to 200 μm, is too big for noninvasive intracellular measurements. Apart from the size, another disadvantage in using optical fibers is that they allow only single-point acquisition and are therefore unsuitable for microscopic imaging. As previously mentioned, ratiometric methods are often preferable for imaging applications and have been applied to measure oxygen concentration inside cells [43].

Different kinds of sensing probes have been developed for extra- and intracellular microscopic imaging such as dendrimers, dye conjugates, and dyes (non)covalently entrapped in polymeric nanoparticles, which have the characteristic of being small enough to be introduced directly into the medium of interest (e.g., blood or interstitial fluid).

When measuring in biological systems, phototoxicity is a potential concern. The byproduct of the quenching reaction is singlet oxygen which is highly reactive and capable of damaging biological tissue. Several solutions to this problem are currently under investigation some of which are already employed, for example the use of PEGylated dendritic “jackets” to regulate the sensitivity and dynamic range of measurements by controlling the oxygen quenching constant [37]. PEG residues are used to modify dendrimers in order to enhance their solubility, to reduce their toxicity, and to help to prevent interactions between the probe and the biological environment. Such probe was tested for high-resolution microscopic in vivo microscopy of vascular pO_2_ in rat’s brain [130]. Another approach consists in the encapsulation of an indicator inside an inert nanoparticle, often referred to as probes encapsulated by biologically localized embedding (PEBBLE) [46]; such nano-spheres can have radii as small as 10 nm and therefore occupy only circa 1 ppb of an average mammalian cell’s volume which has the advantage of causing a rather negligible mechanical perturbation.

Nanosensors have been successfully used to monitor dissolved oxygen in human plasma [34], to monitor cellular respiration [113], and to measure the real-time oxygen concentration inside tumor cells under normal and hypoxic conditions [135].

Nanosensors can be delivered into cells by different techniques such as pico-injectors, gene guns, liposomal incorporation, and endocytosis. Some oxygen nanosensors can spontaneously penetrate cell’s membranes thanks to the presence of positively charged groups on the surface of the particle [69].

Another class of cell-penetrating dyes which rely on an endocytic mechanism of cell entry is represented by the derivative of tetracarboxylic Pt(II)-coproporphyrin I (PtCP) [58, 174]. Such conjugates were successfully tested to measure intracellular O_2_ concentration in live cells, giving information-rich data on cellular function and metabolism. The probe has recently being optimized in order to reduce nonspecific bindings and increase intracellular distribution [57].

Measurements of oxygen concentration in tissue entail additional requirements: the probe does not only have to be highly water soluble, but also to be impermeable to biological membranes so that penetration can be avoided. Another important characteristic is to possess an absorption band in the NIR region since the excitation light needs to penetrate the depths of tissue [130].

Accurate measurements of oxygen concentration in tissue not only give information about tissue oxygenation but also about local microcirculation; such information is of clinical interest for example in radiotherapy and chemotherapy of cancer. Continuous monitoring of oxygen partial pressure has also been realized with the use of an optochemical glass capillary oxygen sensor connected to a microdialysis catheter for the extraction of the biological fluid from a subcutaneous adipose tissue [23].

Two-dimensional pO_2_ distributions were measured over the cross-section of cultivated tissues which were, for this scope, immobilized on top of an optical sensor foil [101]. The experimental setup used by the authors allowed a continuous, noninvasive measurement of tissue oxygen distribution which correlated well with histological analysis and supports the hypothesis that tissue growth in vitro is limited by oxygen supply.

### Marine biology

The accurate measurement of oxygen concentration in fresh and salty water environments has always solicited the interest of scientists in different fields since monitoring the level of DO is essential to clarify several biological processes. Along with oxygen microelectrodes [119], oxygen micro-optodes have been extensively used in marine biology for over a decade [105, 127, 184].

The study of two dimensional (spatial and temporal) distributions and the dynamics of oxygen in marine sediments is also very important, but it cannot be performed with a fiber optic micro-optode since it allows only single-point measurements and it would require a series of sensors (and recording devices) and therefore render the measurement expensive and impractical. Those problems were overcome with the introduction of planar optode to aquatic sciences [85]. Such systems can be based on either intensity (pure or ratiometric) or lifetime-based measurements and they have been developed and applied in many different habitats [121] like microbial mats [84], marine sediments [112, 202], coastal sands [86, 231], permeable sediments [49], rhizospheres [98], and endolithic algal communities [120].

Monitoring of oxygen on 3D surfaces and in tissues is not yet common in marine biology due to the fact that suitable sensing materials (oxygen-sensitive micro- and nanoparticles) became available only in recent years. A very recent work [67] though suggests that such new analytical tools developed by material chemists will become increasingly important in this field in the future. These tools can provide information which is not accessible by more conventional fiber-optic microsensors and planar optodes.

### Enzymatic biosensors

Optical oxygen sensors can be used as transducers for biosensors when coupled with specific biorecognition elements such as enzymes, antibodies, or oligonucleotides [30]. True biosensors can be defined as analytical devices which comprise the following two elements in spatial proximity: a biological element, which is able to interact specifically with the target analyte and a transducer, which transforms the recognition event into a measurable signal. In addition, true biosensors do not need any additional processing steps such as reagent addition and therefore give a reading when exposed to the sample [214].

The most exploited biosensors that make use of optical oxygen sensors as transducer are glucose sensors since they have a wide application in life science, biotechnology, biology, clinical analysis, and food chemistry. In general, the significant interest in sensing glucose is driven by the fact that 4–5 % of the world population suffers from diabetes mellitus which is a complex disorder and its main characteristic is the chronic shift in glucose concentration in blood [210]. There are several complications related to diabetes which are linked to the duration and severity of hyperglycemia (high blood glucose concentration); therefore, it is of extreme importance to maintain the glucose level near to normal values. This can only be provided by a device that continuously measures glucose concentration in the blood. Ideally, the ultimate implantable glucose sensor (used in artificial pancreas) would constantly monitor glucose concentration and automatically activate the release of insulin when needed; unfortunately, such device is still quite far from being fully developed. Many different types of optical glucose sensor have been successfully assembled and used, but since the focus of this review is on optical oxygen sensors, only those which rely on the measurement of oxygen consumption due to the enzymatic oxidation of glucose by glucose oxidase (GOx; Eq. 5) will be described.

5 $$ D-\mathrm{glucose}+{{\mathrm{O}}_{2\ }}\to D-\mathrm{glucono}-1,5-\mathrm{lactone}+{{\mathrm{H}}_{2}}{{\mathrm{O}}_2} $$

One of the first optical biosensor based on GOx immobilized on a nylon membrane was reported in 1988 [218]. Such sensor was based on the quenchability of decacyclene, allowed a continuous determination of glucose in the physiological range and it was in commercial use for more than 10 years. The sensor had been improved years later to obtain shorter response times within the range of 8–60 s [197].

Among the optical devices developed for continuous glucose detection and measurements in vivo, the miniaturized hybrid fiber optical biosensor presented by Klimant and coworkers [181, 182] should be mentioned. The design of this hybrid sensor allows its implantation in subcutaneous tissue and to compensate for the local pO_2_ changes. Two different optodes, one for the determination of glucose and the second one for the determination of local oxygen are placed in close proximity into a polyimide tube; therefore, the final readout which corresponds to glucose can be obtained by the difference between the local oxygen tension (measured by the reference optode) and the reduction of the oxygen level measured by the glucose optode due to the enzymatic reaction [181]. A similar setup has been tested in vitro in a 3-day continuous experiment in glucose-spiked plasma and once coupled with a flow-through cell and commercially available catheter; its ability to measure glucose in humans was also demonstrated in a 24 h test on healthy volunteers [182].

Another fiber-optic dual sensor for the continuous determination of oxygen and glucose was developed by Li et al. [137]. In this approach, the two sensing sites were placed at defined distance between each other on the distal end of an imaging fiber, the changes in fluorescence intensities were therefore captured with a CCD camera. Apart from the instrumental costs, the main drawback of the unit was the dependence of the signal intensity on the dye loading.

Different assemblies of thin film glucose biosensor based on a sol–gel matrix, which are capable of compensating the effect of variable oxygen background, have also been developed by Wolfbeis et al. [236].

Glucose has also been successfully measured online in animal cell cultures with the use of a flow injection analysis system based on fiber optic detection of oxygen consumption using immobilized glucose oxidase [62]. Such system has been tested to be stable for more than 4 weeks in continuous operation withstanding up to 20 analyses per hour and it has been successfully applied to the online monitoring of both glucose and lactate concentrations of an animal cell culture for the production of recombinant human antithrombine III.

Biosensors have also been developed to measure glucose concentration in beverage samples, where the enzyme was either immobilized in eggshell membrane [42] or entrapped in sol–gel [243] and in urine samples where GOx was immobilized in xerogel [240].

Continuous glucose monitoring is of course extremely important, especially for critically ill patients. Many people suffer from diabetes but only one third is aware of it, it is therefore fundamental to have a fast, easy, and cheap way to diagnose diabetes. Wang et al. [230] have developed a new optical biosensor characterized by short response time, lower detection limit, high sensitivity, and stability which can be potentially applied for the fast determination of glucose in human serum. The same group has recently assembled a novel direct readout colorimetric optical glucose sensor strip which can be easily read without instrumental assistance [225]. Such “glucose ruler” was constructed based on a three-layer film which includes a green-emitting CdTe/CdS quantum dots layer as a stable background, a red-emitting platinum–porphyrin oxygen-sensitive layer and a glucose oxidase layer. Oxygen is consumed when the ruler is exposed to glucose and it results in a color change from green to red depending on the concentration.

Biosensors that make use of oxidase type enzyme combined with an optical oxygen transducer have been also designed for other compounds:

- Phenols in hydrophobic organic solvents (enzyme: tyrosinase) [242]
- Alcohols (methanol and ethanol-biosniffer) in water-miscible solvents and in hydrophobic organic solvents (enzyme: alcohol oxidase) [167, 241]
- Cholesterol, for the continuous detection either in aqueous micelle solution or in hydrophobic organic solvents (enzyme: cholesterol oxidase) [239]
- Aspartame in commercial products such as artificial sweeteners (enzymes: α-chymotrypsin and alcohol oxidase) [247]
- Choline-containing phospholipids in serum samples (enzyme: phospholipase-D) [150]
- Bilirubin in serum samples (enzyme: bilirubin oxidase) [136]
- Glutamine in mammalian cells cultures (enzymes: glutaminase and glutamate oxidase) [36]

## Classification of indicators for oxygen sensors

The scope of this review is to give the reader an overview on the state-of-the-art indicators available for optical oxygen sensing. Among the different indicators that have been synthesized and used in this area, one can classify them in two main groups: absorption- and luminescence-based indicators. Absorption-based indicators can be further divided in reversible and irreversible while luminescence-based indicators are only reversible.

## Absorption-based indicators

### Irreversible probes

In general, irreversible absorption-based indicators work by producing a visible color change which is caused by chromogenic chemistry that involves oxidation of the leuco dye by molecular oxygen. Only few of such indicators have been described and their main application can be found in the food and pharmaceutical industries where it is necessary to monitor the headspace gas within packages for meat/fish or for immune reagents, since oxygen is responsible for a variety of food spoiling processes. To be of use for the final consumer, ideal indicators should produce a discernible color change detectable by eyes and should not require specific analytical equipment. Also, an irreversible probe can be preferable to a reversible one in food packaging applications since it can reliably detect the event of the oxygen ingress during the package damage. On the other hand, a reversible probe may still indicate the absence of oxygen in a damaged package since oxygen, after penetration, can be consumed by growing bacterial species.

**Fig. 3:**
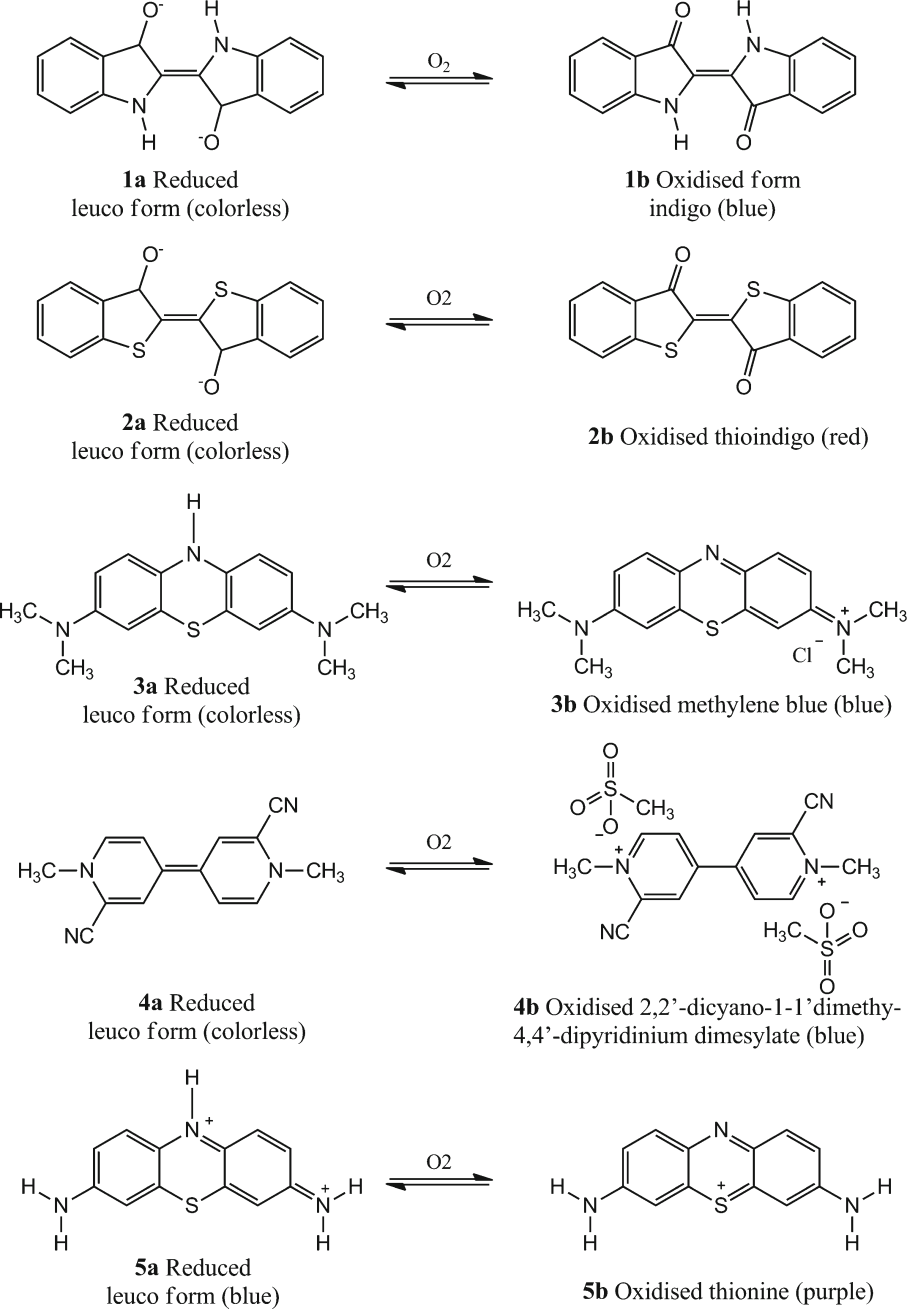
Chemical structures of irreversible probes

Indigo and thioindigo (**1b** and **2b**, respectively, Fig. 3) are vat dyes that can be easily reduced with a suitable reducing agent to become water soluble and colorless. They were incorporated in their reduced form in different polymeric matrices, either moderately or highly oxygen-permeable in order to increase the effective dynamic range [233]. Reaction with oxygen leads to a color change within a few minutes which is quite important when wanting to detect leaks in food packed under modified atmosphere. The authors reported on the possibility to regenerate the sensor immobilized on hydrogel by reduction under inert atmosphere.

Very recently, Mills et al. [162] used a redox dye, methylene blue (MB **3b**, Fig. 3) in combination with a sacrificial electron donor, DL-threitol, and TiO_2_ particles to create an oxygen-sensitive pigment that was then incorporated into a thermoplastic polymer and used as an O_2_ smart plastic film. Once the blue-colored indicator is incorporated in the package, it needs to be activated by UVA light (4 mW cm^−2^) for less than 90 s which causes the MB to be converted to its colorless leuco form that will persist for long time provided that no oxygen is present. It was reported that the indicator can be regenerated in about 4 days. The authors also suggested that the response of the indicator could be increased by depositing Pt nanoparticles onto the TiO_2_ surface.

Methylene blue suffers from a slow reduction to its leuco form and a fast subsequent oxidation by oxygen present at low concentration; therefore, Hay et al. [187] have chosen indicators which showed a much faster reduction after UV exposure, namely 2,2′-dicyano-1-1′-dimethy-4,4′-dipyridinium dimesylate and thionine (**4b** and **5b**, respectively, Fig. 3). This class of indicators shows a moderate sensitivity to oxygen and therefore can be used even when the level increases up to 4 kPa.

### Reversible probes

**Fig. 4:**
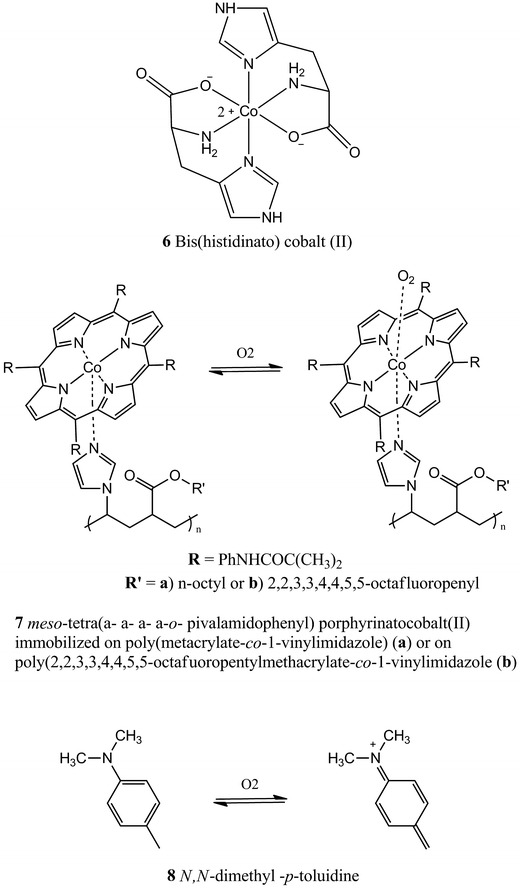
Chemical structures of reversible absorption-based indicators

In our body, oxygen is efficiently transported from the respiratory organs to the rest of the body by hemoglobin, a metallo-protein which binds oxygen reversibly. The association with oxygen generates a shift in the Soret absorption band of hemoglobin; this absorption change was exploited to build an optical sensor to measure pO_2_ from 2.6 to 13.4 kPa, based on immobilized hemoglobin [259]. The main shortcomings of this system are related to the fact that hemoglobin degrades within 2 days when stored at room temperature and 1 week when stored at 4 °C. Therefore, a synthetic, more stable, alternative needed to be found. It is known that some organometallic compounds are able to reversibly bind molecular oxygen [65] and therefore once immobilized on an appropriate support be used as absorption-based oxygen sensors. Among the different organometallic compounds synthesized by Baldini et al. [19], *bis*(histidinato) cobalt(II) [Co(His)_2_] **6** (Fig. 4) was identified to be the best candidate to work as a transducer for oxygen sensing. Such indicator was adsorbed on a thin layer chromatographic plate which was then coated with a layer of silicon rubber; this additional layer is necessary not only to maintain a wet micro-environment but also to mechanically protect the sensitive layer [54]. The sensor was successfully tested in a series of oxygenation/deoxygenation cycles by monitoring the absorption peak at *λ* = 408 nm. It was also found that the response of the indicator is influenced by the pH and it does not give any response at pH lower than 4, since the histidine ligand can exist in different forms depending on pH and a change in the ligand is likely to affect the binding of oxygen.

Another complex that mimics hemoglobin in binding oxygen is the *meso*-tetra(α-α-α-α-*o*-pivalamidophenyl) porphyrinatocobalt(II) **7** [47] which was immobilized on either poly(octylmethacrylate-*co*-1-vinylimidazole) or poly(2,2,3,3,4,4,5,5-octafluoropentylmethacrylate-*co*-1-vinylimidazole) [189]. The complex in the presence of oxygen forms an oxo-adduct (Fig. 4) at the cobalt center which causes a shift in the absorption spectra, monitored at 547 nm. The sensing membranes are stable for 1–2 months and possess a useful response range between 0.1 and 100 % of oxygen at atmospheric pressure.

A fiber optic oxygen sensor for gaseous O_2_ measurements was successfully developed by Choi and Hawkins [40, 41]. The working principle of this sensor is based on the contact charge–transfer absorption of *N*,*N*-dimethyl-*p*-toluidine **8** (Fig. 4) at 400 nm. The indicator is entrapped inside a poly(tetrafuoroethylene) tubing and inserted into a glass flow though cell. The described sensor can detect O_2_ from 4.3 to 100 %, it uses very inexpensive materials and its fabrication is quite simple and fast but it suffers from several drawbacks such as interference from Cl_2_ and SO_2_, long response time and recovery times (12–26 min).

None of the absorption-based systems presented above became widespread in practice since the resolution provided is quite low as the spectral changes upon oxygen binding are relatively small. It should be mentioned here the existence of another technique for absorption-based oxygen sensors that relies on monitoring of the lifetime change of the triplet–triplet (*T*_1_ − *T*_*n*_) absorption [3]. The triplets of the indicators produced during excitation are dynamically quenched by molecular oxygen. Theoretically, the number of oxygen indicators can be greatly extended since any luminescent or nonluminescent molecule possessing efficient intersystem crossing is suitable. In practice though, the use of this technique is severely limited by the need of a quite complicated setup and high power light sources to allow an efficient population of triplet states. Two classes of indicators have been reported so far, Zn^2+^ porphyrins, which have a long lifetime of photoexcited triplet state (~100 μs) and fullerene (C_60_ and C_70_) which have a longer photoexcited triplet state (~20 ms) that is efficiently quenched by oxygen [3].

## Luminescence-based indicators

To date, the most common and useful types of optical oxygen sensors are those based on the quenching of luminescence of appropriate indicators by molecular oxygen. The ability of oxygen to decrease the luminescence of dyes adsorbed on inorganic surfaces was first observed and published by Kautsky back in 1939 [100] who immobilized organic dyes such as trypaflavin, chlorophyll, porphyrins, eosin, erythrosine on either solid silica gel or aluminum oxide gel and observed that both the fluorescence and the phosphorescence of the adsorbates are reversibly quenched by oxygen.

Since then, different analytical applications of luminescence quenching for the determination of oxygen partial pressure have been reported. In 1944, Pollack et al. [185] described a method to measure oxygen production by exploiting the quenching of phosphorescence of an unspecified dye adsorbed in silica gel. Shaw [204] applied the ability of O_2_ to quench the phosphorescence emission of phenanthrene and triphenylene immobilized in acrylic media as a method to measure the diffusion coefficients of oxygen through the polymeric media.

Jones [99] determined the oxygen permeability in thin acrylic films by measuring the quenching rate of the phosphorescence lifetime of naphthalene which was added to the film as an additive. A prototype to measure the atmospheric oxygen tension based on the quenching of fluorescence of fluoranthene adsorbed on porous glass was developed by Bergman in 1967 [22].

The number of luminescent indicators synthesized and exploited in optical oxygen sensing has of course largely increased as researchers started recognizing their potential to measure oxygen concentration in different fields of science as previously described.

The reader will now find the description of luminescent indicators classified in the following groups: polycyclic aromatic hydrocarbons, polypyridyl complexes, metal porphyrins, cyclometallated complexes, and complexes with rarely used central atoms. Indicators for oxygen sensing not belonging to any of these categories can be found in the section called “Miscellaneous”.

1. Polycyclic aromatic hydrocarbons

**Fig. 5:**
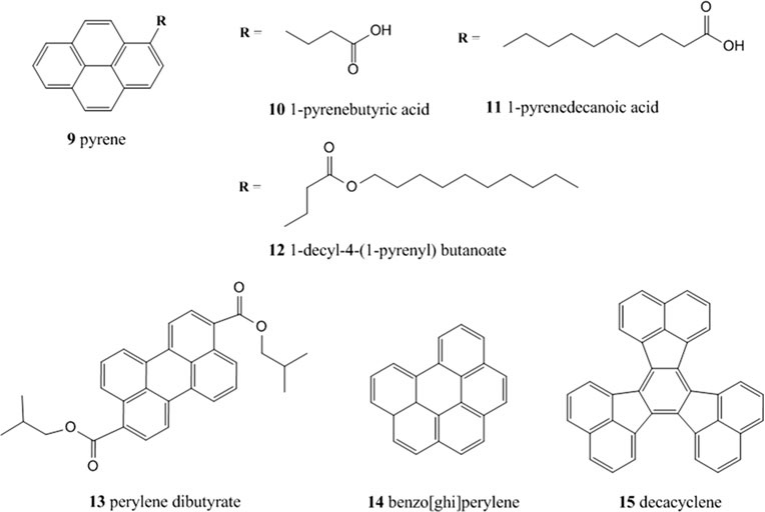
Chemical structures of polycyclic aromatic hydrocarbons

Polycyclic aromatic hydrocarbons (PAHs), especially pyrene and its derivatives (Fig. 5), have been used as fluorescent indicators as they possess a relative long excited-state lifetimes and can be quenched by oxygen in the 0–40 kPa range (Table 1).

**Table 1:** Photophysical properties of polycyclic aromatic hydrocarbons

Dye	*λ*_max_^abs^ (nm)	λ_max_^em^ (nm)	Medium	*Φ*	*τ* (ns)	References
**9** Pyrene	305/319/335	381/390	Cyclohexane	0.65	450	[252]
**10** 1-Pyrenebutyric acid	365	376/396/447	Toluene	–	200	[3, 176]
**11** 1-Pyrenedecanoic acid	340	376/396	Alumina plate			[78]
**12** 1-Decyl-4-(1-pyrenyl) butanoate	345	480	Toluene	–	–	[21]
**13** Perylene dibutyrate	468	514	Toluene	–	–	[183]
**14** Benzo[ghi]perylene	386	419	Methanol	0.26^a^	110	[97]
**15** Decacyclene	385	510	Toluene	0.29	–	[218, 237]

^a^Taken from [53]

Pyrene (**9**) has a relatively high quantum yield (*Φ*) of circa 0.7, good pressure sensitivity, and low temperature coefficient at ambient temperature; characteristics that can make it a suitable fluorophore for pressure sensitive paint (PSP). On the other hand, studies have demonstrated lack of stability of this indicator which tends to diffuse out of the support and evaporate [20]. Also, low solubility of planar PAHs and their tendency to aggregate in polymer represent another hindrance. Therefore, a number of pyrene derivatives bearing long lipophilic chains were prepared to overcome the above disadvantages. For example, Basu et al. [21] synthesized a pyrene derivative: 1-decyl-4-(1-pyrenyl) butanoate **12** which has a high oxygen quenching sensitivity and lower diffusion coefficient in silicone polymer. Perylene dibutyrate **13** has been incorporated in an organic polymer adsorbent (amberlite) and deposited on an optical fiber for in vivo oxygen concentration measurements [183].

Among the different PAHs, 1-pyrenebutyric acid **10** possesses longer fluorescence lifetime (0.2 μs) and therefore it is suitable for optical oxygen sensing devices; it has been demonstrated that isolated rat liver cells easily take up 1-pyrenebutyric acid in concentrations up to 0.8 mM and the fluorescence probe can be used to measure intracellular oxygen concentration [108]. In general, PAHs are not very soluble in polymeric matrices; to overcome this problem, Amao et al. [13] fabricated a probe for oxygen sensing based on 1-pyrenebutyric acid adsorbed on the surface of an alumina plate.

1-Pyrenedecanoic acid **11** can also be chemisorbed onto alumina plate through its carboxyl group and the resulting sensor film has rapid response and recovery times. The fluorescence lifetime of benzo[ghi]perylene **14** is longer than 100 ns and its absorption maximum coincided with the wavelength of the second-harmonic emission from a near-infrared semiconductor laser which was used as the light source in an optical fiber setup for the determination of oxygen concentrations [175].

Decacyclene **15** has been used for more than 10 years as the oxygen indicator in a glucose biosensor [218] and it was the indicator of choice in a dual sensor for oxygen and halothane [237]; it can be excited by a blue LED and it has the advantage of not producing singlet oxygen when quenched.

This class of dyes in general suffers from many disadvantages which has limited their application as indicators for oxygen sensors. They have short excitation wavelength (300–390 nm) which causes components of biological media, optical components or polymers to display strong background fluorescence which is emphasized by small Stokes’ shifts; despite good emission quantum yields the molar absorption coefficients are rather low which results in moderate brightness; the fluorescence decay times do not exceed several hundred nanoseconds so that sufficient sensitivity can be achieved only in highly gas-permeable polymers; finally, the majority of PAHs is not well soluble in polymeric matrices and therefore suffers from lack of stability due to aggregation.

It can be summarized here that PAHs and other fluorescent oxygen indicators which have been of importance in the early stage of sensor development are nowadays almost completely substituted by more advanced indicators which will be described below.

2. Transition metal polypyridyl complexes

Many luminescent transition metal polypyridyl complexes have been synthesized and some have been used as indicators for oxygen sensing. Within this group of indicators, transition metals used are Ru(II) and Os(II). In general, their complexes are characterized by large Stokes’ shifts, possess excitation and emission maxima in the visible region, show good photostability, and their luminescent properties can be tuned as a function of the ligands.

**Fig. 6:**
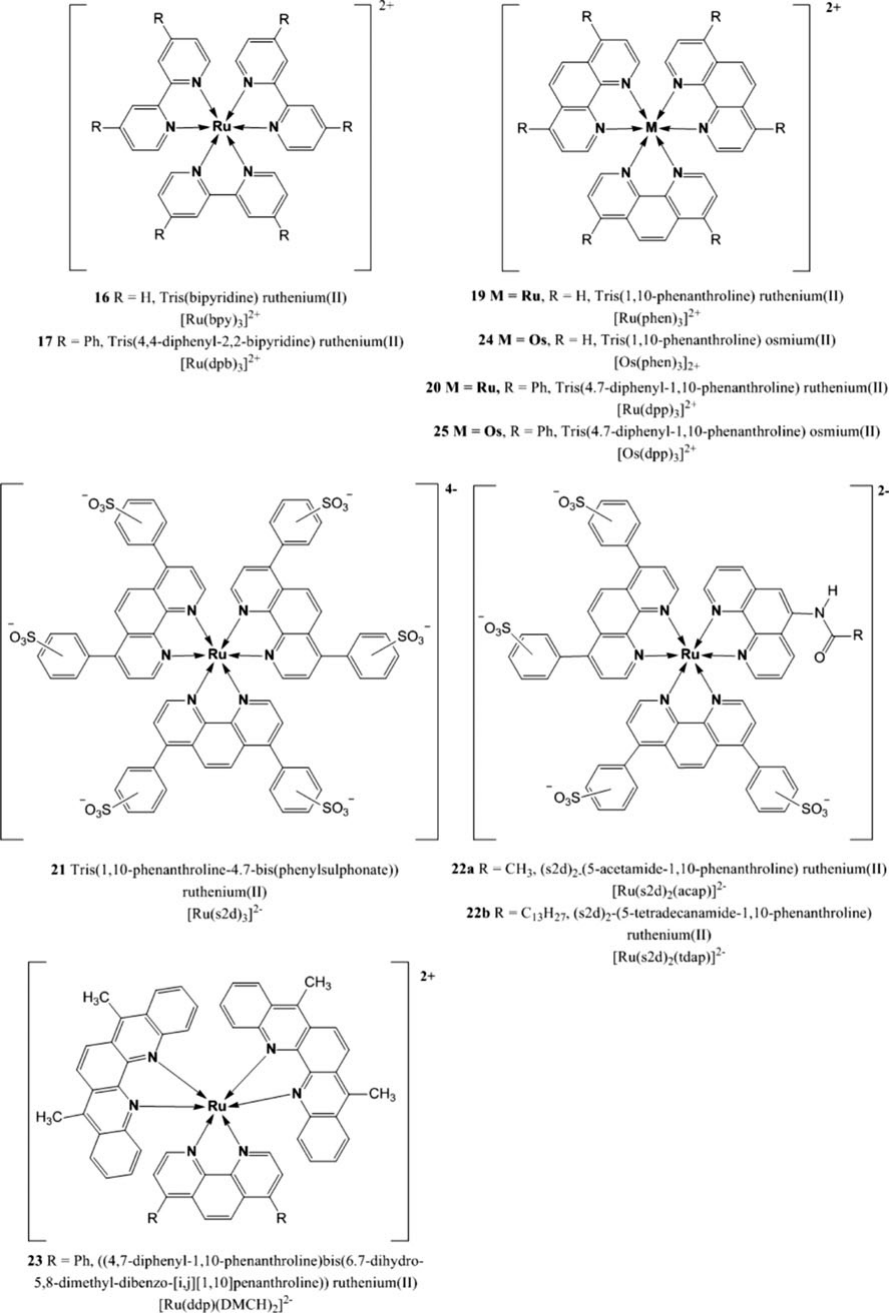
Chemical structures of Ru(II) and Os(II) polypyridyl complexes

Within this group, Ru(II) polypyridyl complexes have been used more frequently due to their relatively high brightness and relatively long lifetimes; this is especially true for the *tris*(4,7-diphenyl-1,10-phenanthroline) ruthenium(II) complex [Ru(dpp)_3_]^2+^ **20** (Fig. 6) which has a luminescence lifetime up to 6.4 μs and a quantum yield of 0.3 [2]. Despite the disadvantages which will be described below, this dye is still one of the most popular oxygen indicators in use.

The luminescence of Ru(II) polypyridyl complexes is dominated by the metal to ligand charge transfer (MLCT) process that involves the promotion of an electron from a metal *d* orbit to a ligand π^*^ orbit; and therefore has been shown to be dependent on the polarity of the microenvironment and on the donor/acceptor character of the surrounding medium because of the significant changes produced on the electronic charge distribution of the molecule upon photoexcitation [166]. Also, both the excitation and the emission bands of the MLCT complexes are very broad which can be viewed as a positive property (higher flexibility in the choice of the excitation sources) but can also be a disadvantage (lower molar absorption coefficients and difficulties in isolating the emission in multi-analyte systems).

Luminescence lifetimes of several microseconds result in rather low oxygen sensitivity in common polymeric matrices such as polystyrene [91]; the resolution of such sensors in the range of 0–21 kPa O_2_ is often not sufficient for practical applications. To overcome this problem, the ruthenium(II) polypyridyl complexes have been immobilized in highly oxygen-permeable matrices such as silicon rubbers or organically modified silica (Ormosil). Unfortunately, [Ru(dpp)_3_]^2+^, just like the majority of the other ruthenium polypyridyl complexes, is ionic and therefore it displays very poor solubility in silicon rubber which is one of the most common matrices for optical oxygen sensors as it has a high permeability for oxygen, good adhesion to glass fibers, inertness to biological samples, optical transparency, and excellent chemical and mechanical stability. Klimant and Wolfbeis [107] replaced the inorganic counter ion with an organic one to render the dye silicone soluble. Dodecyl sulfate (*n*-C_12_H_25_SO_3_^−^/DS) or trimethylsilylpropanesulfonate ((CH_3_)_3_SiCH_2_CH_2_CH_2_SO_3_^−^/TSPS) were used. They demonstrated that, thanks to the enhanced solubility, the modified indicators could be used as indicators for oxygen sensors in high concentrations so that very thin but highly luminescent layers could be prepared.

In the same way, Mills [164] incorporated the fluorophore cation into a variety of different plasticized polymeric films (e.g., polymethylmethacrylate (PMMA)/tri-*n*-buthylphospate (TBP)) using a tetraphenylborate anion.

Alternative matrices have been used to immobilize [Ru(dpp)_3_]^2+^ to construct a feasible optical oxygen sensor. Examples are sol–gel [147, 152, 154, 249], ethyl cellulose or PVC membranes [61], different types of ORMOSILs [39, 94, 106], polystyrene [91], polysulfones [11, 18], poly(dimethylsiloxane) alone [251] or with different amounts of pendant acrylate groups [168], and a blended fluoropolymer matrix consisting of Nafion^®^ and Aflas^®^ [83]. In another approach, the [Ru(dpp)_3_]^2+^ complex was adsorbed onto silica and then dispersed in a silicone rubber support [38, 93]. Finally, the ruthenium indicator can be cross-liked with isobutyl methacrylate and 2,2,3,3-tetrafluoropropyl methacrylate to obtain a porous plastic probe which can be easily coupled with common optic fibers [87].

Optical oxygen sensors have been developed using various Ru(II) polypyridyl complexes such as [Ru(bpy)_3_]^2+^ **16** (bpy = bipyridyl) [142, 159, 235], [Ru(dpb)_3_]^2+^ **17** (4,4-diphenyl-2,2-bipyridine) [232], [Ru(5-odap)_3_]^2+^ **18** (5-odap = 5-octadecanamide-1,10-phenanthroline) [166], [Ru(phen)_3_]^2+^ **19** (phen = phenanthroline) [227], [Ru(s2d)_3_]^4−^ **21** (s2d = 1,10-phenanthroline-4,7-*bis*(phenylsulfonate)) also modified with 5-acetamide-1,10-phenanthroline (acap) **22a** or 5-tetradecanamide-1,10-phenanthroline (tdap) **22b** [245] (Fig. 6, Table 2). These indicators have not been as widely used as [Ru(dpp)_3_]^2+^ due to their lower quantum yield and shorter luminescent lifetime.

**Table 2:** Photophysical properties of transition metals polypyridyl complexes

Dye	λ_max_^abs^ (nm; ε × 10^−4^ M^−1^ cm^−1^)	λ_max_^em^ (nm)	Medium	*Φ*	*τ*	References
**16** [Ru(bpy)_3_]^2+^	450 (1.43)	630	EtOH-MeOH (4:1 *v*/*v*)	0.089	1.15 μs^a^	[48]
**17** [Ru(dpb)_3_]^2+^	473 (2.80)	635	EtOH-MeOH (4:1 *v*/*v*)	0.306	1.95 μs	[48]
**18** [Ru(5-odap)_3_]^2+^	449 (1.75)	600	MeOH	0.027	0.50 μs	[166]
**19** [Ru(phen)_3_]^2+^	444 (2.00)	596	MeOH	0.019	0.28 μs	[166]
**20** [Ru(dpp)_3_]^2+^	463 (2.86)	618	EtOH-MeOH (4:1 *v*/*v*)	0.366	6.40 μs	[2]
**21** [Ru(s2d)_3_]^4-^	475	620	MeOH	–	0.31 μs	[245]
**22a** [Ru(s2d)_2_(acap)]^2-^	475	616	MeOH	–	0.32 μs	[245]
**22b** [Ru(s2d)_2_(tdap)]^2-^	475	605	MeOH	–	0.30 μs	[245]
**23** [Ru(ddp)(DMCH)_2_]^2+^	563	738	EtOH	0.009	650 ns	[103]
**24** [Os(Phen)_3_]^2+^	432/478/660	691	CH_2_Cl_2_	–	6.0 ns	[250]
**25** [Os(dpp)_3_]^2+^	454/500/580/650	729	CH_2_Cl_2_	–	4.6 ns	[250]
**26** [Os(bpy)_2_(Nbpy)]^2+b^	490	800	Water	0.0096	12 ns	[155]
**27** [Os(dpp)_2_(Nbpy)]^2+^	450	795	Water	0.013	15 ns	[155]
**28** [ReL(CO)_3_CNR]^+c^	**a** 338 (0.43)	**a** 448	CH_2_Cl_2_	**a** 0.59	**a** 1.97 μs	[194]
	**b** 386 (0.7)	**b** 496		**b** 0.60	**b** 44.4 μs	
**29** Pt(ddp)(CN)_2_	293 (41.3)/375 (5.9)/357 (7.5)	630	Polyethylene glycol	–	1 μs	[122]

^a^Taken from [141]

^b^Nbpy = *N*-(6-aminohexyl)-4′-methyl-2,2′-bipyridine-4-carboxamide

^c^L = 2,2-bipyridine (**a**) or 1,10-phenanthroline (**b**) and R = *tert*-butyl

In general, one advantage of Ru(II) polypyridyl complexes is that they are fairly easy to prepare, reason why they are quite popular as indicators for early optical oxygen sensors; on the other hand, though they present many disadvantages they show only moderate molar absorption coefficients and therefore brightness, they possess relatively short lifetimes, their absorption and emission spectra are usually very broad which implies the use of specific filters and more expensive instrumentation and they suffer from pronounced cross talk to temperature since their triplet states are subjected to severe thermal quenching [140].

The absorption of the of Ru(II) complexes is located in the blue part of the spectrum (Table 2) which can be a severe limitation for many applications (particularly for in vivo measurements); thus, Klimant et al. [103] used Ru(II) complexes based on π-extended polypyridyl ligands ([Ru(ddp)(DMCH)_2_]^2+^ **23**), whose absorption and emission bands were bathochromically shifted of about 100 nm compared to the conventional Ru(II) complexes. Unfortunately, the brightness of the new indicators was too low (*Φ* did not exceed 1 %) and the decay times too short (<700 ns).

In an alternative approach, Demas et al. [250] focused their attention on Os(II) complexes which are analogous to the Ru(II) but can be excited in the red part of the spectrum and are therefore compatible with red LEDs and low-cost red lasers diodes. It has also been argued that the Os(II) complexes should be even more photostable and robust than the Ru(II) complexes as they present a larger energy gap between the emitting state and the photochemically destructive upper *dd* states [109]. Optical oxygen sensors have been developed using mainly two indicators: [Os(phen)_3_]^2+^ **24** and [Os(dpp)_3_]^2+^ **25** (Fig. 6) immobilized in poly(dimethylsiloxane) (PDMS) and in Gp-163 (an acrylate-modified PDMS). The luminescence lifetimes of such indicators in dichloromethane are in the nanosecond range which is much shorter when compared to the Ru(II) analogs; thus, only very highly oxygen-permeable materials can be adequate to obtain measureable responses. It should also be mentioned here that the luminescence quantum yields of the Os(II) are much lower than for the Ru(II) analogs (Table 2) which is a severe disadvantage.

**Fig. 7:**
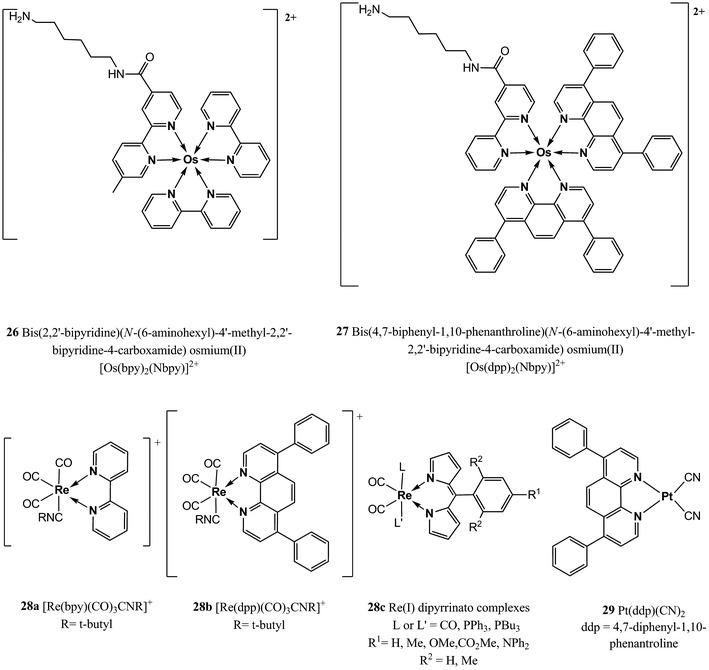
Chemical structures of Os(II), Re(I), and Pt(II) polypyridyl complexes

Other two osmium polypyridyl complexes have been used by Bawendi et al. [155] in conjunction with nanocrystals as two-photon oxygen sensors for application in biological microenvironments; namely *bis*(2,2′-bipyridine)(*N*-(6-aminohexyl)-4′-methyl-2,2′-bipyridine-4-carboxamide) osmium(II), [Os(bpy)_2_(Nbpy)]^2+^ **26** and *bis*(4,7-diphenyl-1,10-phenanthroline)(*N*-(6-aminohexyl)-4′-methyl-2,2′-bipyridine-4-carboxamide) osmium(II), [Os(dpp)_2_(Nbpy)]^2+^ **27** (Fig. 7). The free amine groups of the modified polypyridynes were linked to a carboxylic acid functionality of a semiconductor nanocrystal (NC) to afford a biologically stable amide bond; the resulting compounds possess absorption profiles that extend well into the red spectral region, leading therefore to a good overlap with the emission band of the NCs donors and allowing fluorescence resonance energy transfer from the semiconductor to the osmium complex. Since the NC emission is insensitive to oxygen, it can be used as an internal reference while the enhanced Os(II) emission can be exploited for sensing. However, the aforementioned disadvantages of the Os(II) complexes are also valid for these indicators.

Also, Re(I) complexes have been used as oxygen indicators, however, their structure is different from the Ru(II) and Os(II) complexes since they contain only a single polypyridyl ligand and additional carbonyl ligands are necessary to obtain sufficient luminescence. [Re(bpy)(CO)_3_CNR]^+^ **28a** and [Re(dpp)(CO)_3_CNR]^+^ **28b** (Fig. 7) can be mentioned as an example. These dyes possess very high quantum yields (~0.7) and long lifetimes (>40 μs) but they lack significant absorption above 400 nm and their molar absorption coefficients are quite low (<7,000 M^−1^ cm^−1^) [193, 194].

Recently, Telfer et al. [156] reported the first Re(I)-dipyrrinato complexes (**28c**) which in comparison with **28a** and **b**, have higher molar absorption coefficients (2.5–4.2 × 10^4^ M^−1^ cm^−1^) but much lower quantum yields (>0.01; Fig. 7).

Even though platinum complexes do not need extra ligands to increase their luminescence, a Pt(II) complex with a structure similar to **28** has been reported. Pt(ddp)(CN)_2_ **29** is of interest since it is very soluble and stable in silicone rubber and shows no photochemical degradation (photophysical properties are shown in Table 2) [122]. Complex **29** being neutral can be mixed with silicone rubber quite easily and it does not leach into aqueous solutions. Moreover, Pt(ddp)(CN)_2_ forms excimer upon irradiation and its emission can be easily monitored, therefore **29** can be used as spectroscopic probe of microenvironments inside polymers.

3. Metalloporphyrins

Among all the indicators that have been used for optical oxygen sensors, Pt(II) and Pd(II) porphyrins are the most popular luminophores since they possess strong phosphorescence at room temperature, moderate to high molar absorption coefficients and large Stokes’ shifts. Additionally, phosphorescence lifetimes are rather long (microsecond to millisecond) and can be tuned by varying the nature of the central atom. As it can be noted from Table 3, in general, for each pair of metalloporphyrin complex the one in which *M* = Pd(II) shows much longer lifetimes with respect to the Pt(II) analog, this can be related to the increase in spin-obit coupling expected in the heavier metal [64]; on the other hand, platinum(II) complexes possess approximately two to three times higher emission quantum yields than the respective palladium(II) derivatives.

**Table 3:** Photophysical properties of most common metalloporphyrin oxygen indicators

Dye	*λ*_max_^abs^ (nm) (ε × 10^−3^ M^−1^ cm^−1^)	*λ*_max_^em^ (nm)	Medium	*Φ*	*τ*	References
**30** PtOEP	382/536	649	Toluene	0.41	75 μs	[115]
**31** PdOEP	546	670	PS	0.20	990 μs	[177]
**32** PtTFPP	390 (323)/504 (23.2)/538 (29.4)	647/710	CH_2_Cl_2_	0.088	60 μs	[123]
**33** PdTFPP^a^	406 (192)/519 (18.2)/552 (15.5)	738	CH_2_Cl_2_	0.21	1.65 ms	[209]
**34** PtTCPP	402 (210)/514 (21)	675	Water	0.01	34 μs	[222]
**35** PdTCPP	418/523/556	700	DMF	–	–	[4]
**36** PtOEPK^b^	398 (86.2)/592 (55.1)	758	CHCl_3_	0.12	60 μs	[179]
**37** PdOEPK^b^	410 (82.6)/603 (53.5)	789	CHCl_3_	0.01	455 μs	[179]
**38** PtTFPPL	396 (180)/574 (65.5)	733	3-Methyl pentane	–	72 μs	[102]
**39** PdTFPPL	412 (120)/584 (39.2)	758	3-Methyl pentane	–	0.8 ms	[102]
**40** PtTPTBP	430 (205)/614 (136)	770	Toluene	0.51	47 μs	[27]
**41** PdTPTBP	443 (416)/628(173)	800	Toluene	0.21	286 μs	[27]
**42** PtTPTBPF	430 (212)/615 (146)	773	Toluene	0.60	50 μs	[27]
**43** PdTPTBPF	443 (268)/629 (115)	803	Toluene	0.23	297 μs	[27]
**44** Pt1NF	434 (272)/628 (147)/638 (140)	815	Toluene	0.53	44 μs	[172]
**45** Pd1NF	450 (202)/641 (74.0)/654 (72.6)	849	Toluene	0.18	203 μs	[172]
**46** Pt2NF	438 (106)/652 (108)	835	Toluene	0.27	28 μs	[172]
**47** Pd2NF	452 (190)/608 (102)/666 (142)	868	Toluene	0.12	138 μs	[172]
**48** Pt3NF	441 (108)/635 (21.9)/667 (83.5)/678 (78.9)	870	Toluene	0.25	21 μs	[172]
**49** Pd3NF	456 (138)/630 (17.0)/652 (22.0)/681 (63.8)/691 (60.0)	~882	Toluene	0.07	106 μs	[172]
**50** PtTPTNP	436/689	883	Toluene	0.22	8.5 μs	[207]
**51** PdTPTNP	465/721	948	Pyridine	0.079	63 μs	[72]
**52** PtTBP(CO_2_Bu)_8_	416 (170)/609 (166)	745	Pyridine	0.51	50 μs	[73]
**53** PdTBP(CO_2_Bu)_8_	426 (302)/618 (158)	770	DMF	0.23	400 μs	[73]
**54** PdPh_4_TBP (CO_2_Me)_8_	460 (251)/636 (87)	787	DMF	0.03	62 μs	[73]
**55** PtNTBP	406 (106)/606 (61)/630 (137)	844	Toluene	0.22	40 μs	[31]
**56** PdNTBP	421 (106)/619 (58)/642 (133)	875	Toluene	0.08	213 μs	[31]
**57** PtN_2-cis_TBP	388 (51)/621 (147)	841	Toluene	0.17	20 μs	[31]
**58** PdN_2-cis_TBP	380 (33)/631 (98)	873	Toluene	0.05	101 μs	[31]
**61** Oxyphor R2	415/524 (19)	690	Water	0.10	771 μs^c^	[63]
**62** Oxyphor G2	442/632 (50)	790	Water	0.12	280 μs^c^	[63]
**63** PdTBP-(AG^2^PEG)_8_	446/635	816	Phosphate buffer	0.03	270 μs	[130]
**64** IrOER-CO-Cl	404 (165)/518 (15)/550 (31)	672	Toluene	0.14	97 μs	[115]
**65** Ir-OEP-Py_2_	389 (148)/509 (11)/539 (26.6)	655	Toluene	0.195	40 μs	[115]
**66** Ir-OEP-*n*-butIm_2_	390 (150)/508 (9.7)/541 (15)	655	Toluene	0.20	27 μs	[115]
**67** IR-OEP-CarbIm_2_	388 (142)/507 (10)/538 (18)^h^	652^d^	Toluene	0.21^d^	37 μs^e^	[115]

^a^Emission spectra Φ and lifetimes were measured in 3-methylpenthane at 77 K

^b^Emission spectra Φ and lifetimes were measured in a sodium sulfite solution

^c^Measured in deoxygenated condition at 23.5 °C and pH = 6.4

^d^In polystyrene at 25 °C

^e^Measured in EtOH

A large number of Pt(II) and Pd(II) pophyrin complexes have been synthesized and many have been modified during the years to improve the photophysical properties such as brightness, lifetime, photostability, and to tune the wavelength of absorption, in order to make the indicators suitable for oxygen sensing in different conditions and media.

Platinum and palladium complexes with octaethylporphyrin (PtOEP **30**, PdOEP **31**) remain popular indicators due to their strong room temperature phosphorescence with quantum yields of about 0.5 and 0.2, respectively, and long lifetimes under anoxic conditions (ca. 91 μs and ca. 990 μs, respectively) [177]. These indicators have been widely used as optical oxygen sensors and have been immobilized in various oxygen-permeable polymeric matrices such as polystyrene [104, 178], sol–gels [132, 133], poly(aryl ether ketone) [14], ethyl cellulose, cellulose acetate butyrate and polyvinylchloride [60], styrene-pentafuorostyrene copolymer film [9], and poly(1-trimethylsilyl-1-propyne) [6]. A general disadvantage of these indicators is their rather low photostability.

Platinum tetrakis(pentafuorophenyl)porphyrin (PtTFPP **32**) has received much attention and mostly replaced PtOEP because it shows much less photodeterioration [186]. The higher photostability of **32** compared to **30** is explained by the electron-withdrawing character of the perfluorophenyl substituents which reduces the electron density on the porphyrin ring thus rendering the PtTFPP less reactive toward oxidation by singlet oxygen [134]. PtTFPP possesses similarly long decay time (60 μs) and acceptable brightness when excited in the visible rage; it has been widely used as oxygen indicator in different types of matrices such as polystyrene [5, 134], fluoroacrylic polymer as a component of a pressure-sensitive paint [51, 186], in sol–gel [44, 45], poly(norobornene)s [213], and many other matrices. PtTFPP was also modified to be able to be polymerized and cross-linked with hydrophilic poly(2-hydroxyethyl methacrylate)-co-polyacrylamide or hydrophobic polystyrene to generate highly photostable and biocompatible sensing films [216]. Nucleophilic substitution of the *para*-fluorine atoms of the pentafuorophenyl groups allows covalent coupling to polymeric materials such as polystyrene copolymers [114] and silica gel [25]. Due to its excellent photostability, PtTFPP has been extensively used for those applications in which high light intensities are used, primary in fiber-optic microsensors and microscopy [69], but also in multi-analyte sensors and as components for pressure-sensitive paints [76].

The Pd(II) analogue **33** is characterized by a rather long lifetime (~1 ms at room temperature); sensors based on this complex covalently attached to the surface of amino-modified silica–gel particles and dispersed in silicon rubber, show higher sensitivity with a dynamic range from 0.02 to 100 Pa and therefore can be used to measure trace level of oxygen. Both PtTFPP and PdTFPP were covalently immobilized on amino-functionalized silica–gel particle to obtain heterogeneous sensing materials which possess high photostability and fast response time [25].

Platinum and palladium tetrakis(4-carboxyphenyl)porphyrin (PtTCPP **34** an PdTCPP **35**) do not possess very high quantum yields or long lifetimes; nevertheless, several sensors using these compounds have been reported [4, 10, 146]. The metal complexes can be chemisorbed on alumina because of the formation of a stable bond between alumina and the carboxyl group of the TCPP.

More exotic oxygen sensors relied on platinum(II) and palladium(II) complexes with cationic water-soluble porphyrins which were electrostatically immobilized in the negatively charged fluorinated Nafion^®^ membrane [221]. The sensors were shown to be suitable for measurements of oxygen in gas phase but their performance in aqueous solutions, evidently, may be compromised by ionic species.

The brightness (defined as the product of the molar absorption coefficient ε and the quantum yield *Φ*) of the Pt(II) and Pd(II) complexes with OEP and TFPP is very good upon excitation in the Soret band (UV/violet light) due to high molar absorption coefficients for this transition (Table 3). However, excitation in the Q bands located in the green part of the spectrum (which is often more preferable) is about tenfold less efficient. Therefore, rather thick sensing layers are necessary to insure sufficient absorption and a good S/N ratio. This results in slower dynamic response of the sensors. Also, if used in the form of oxygen-sensitive nanoparticles higher concentration of those is required and this can disturb biological systems. To overcome this drawback, Mayr and co-workers proposed an approach to enhance the brightness of oxygen (and other optical sensors) via light harvesting [151]. Here, excitation is performed via an antenna dye which is added along with an oxygen indicator; the latter becomes excited via the Föster Resonance Energy Transfer.

The indicators described so far efficiently absorb light only in the UV–vis region and therefore suffer from some drawbacks: (1) high level of autofluorescence is produced upon excitation in the UV–vis since many natural compounds which are present in biological samples, such as nucleotides FAD and NAD, are fluorescent, (2) they are poorly suitable for measurements in scattering media such as marine sediments or tissues, (3) they cannot be used in implantable sensors since blood efficiently absorbs in visible region.

Excitation in the red part of the spectrum is strongly preferred not only because it allows sensing in highly scattering media and in tissue but also because it can be combined with cheaper optical components; for example, the sensitivity of cheap and compact Si photodiodes increases at wavelengths longer than 600 nm and reaches a maximum at 850–900 nm [27]. For these reasons, red-excitable and NIR-emitting indicators are becoming increasingly popular for the development of optical oxygen sensors to be used in marine sediments, in blood, in cells, or in tissues.

Compared to the metalloporphyrins described above, platinum(II) and palladium(II) complexes with porphyrin ketones (PtOEPK **36**, PdOEPK **37**) and porphyrin lactones (PtTFPPL **38**, PdTFPPL **39**) possess bathochromically shifted absorption peaking at 570–600 nm. Unfortunately, these dyes have only moderate emission in the NIR (Table 3).

The Pt(II) and Pd(II) porphyrin ketones which were obtained via oxidation of the porphyrin macrocycle and displayed high photochemical stability [177]. PtOEPK and PrOEPK display room temperature phosphorescence with reasonable quantum yield, particularly the Pt(II) complex (*Φ*_*P*_ > 0.1), and long lifetimes (60 μs for the Pt(II) and 455 μs for the Pd(II) complexes) but only moderate to low brightness; they have been used in different matrices such as polystyrene and polyvinylchloride which influenced the dynamic range of the sensors [104, 111, 217].

Recently, Nock et al. [173] applied the platinum(II) octaethylporphyrinketone/polystyrene probe for measurements of both gaseous and dissolved oxygen in microfluidic devices. Platinum(II) and palladium(II) tetra(pentafluorophenyl)porpholactone are similar in their spectral properties to the complexes of porphyrin–ketones and show phosphorescence lifetimes that are about 60 % that of the unmodified TFPP complexes (~70 μs for PtTFPPL and ~1 ms for PdTFPPL). The lactone ring was produced from the parent free-base porphyrin via oxidation and it is responsible for a shift in the emission spectra toward longer wavelengths; also, the lactone functionality makes this indicators even more stable toward photo-oxidation [102].

Palladium(II) and platinum(II) complexes with benzo- [188] and naphthoporhyrins [73, 188, 191] represent another group of the NIR oxygen indicators. These dyes belong to the class of π-extended porphyrins which are characterized by an extended aromatic fragment fused on the porphyrin core; this feature is responsible for the bathochromic shift of the absorption and emission bands.

**Fig. 8:**
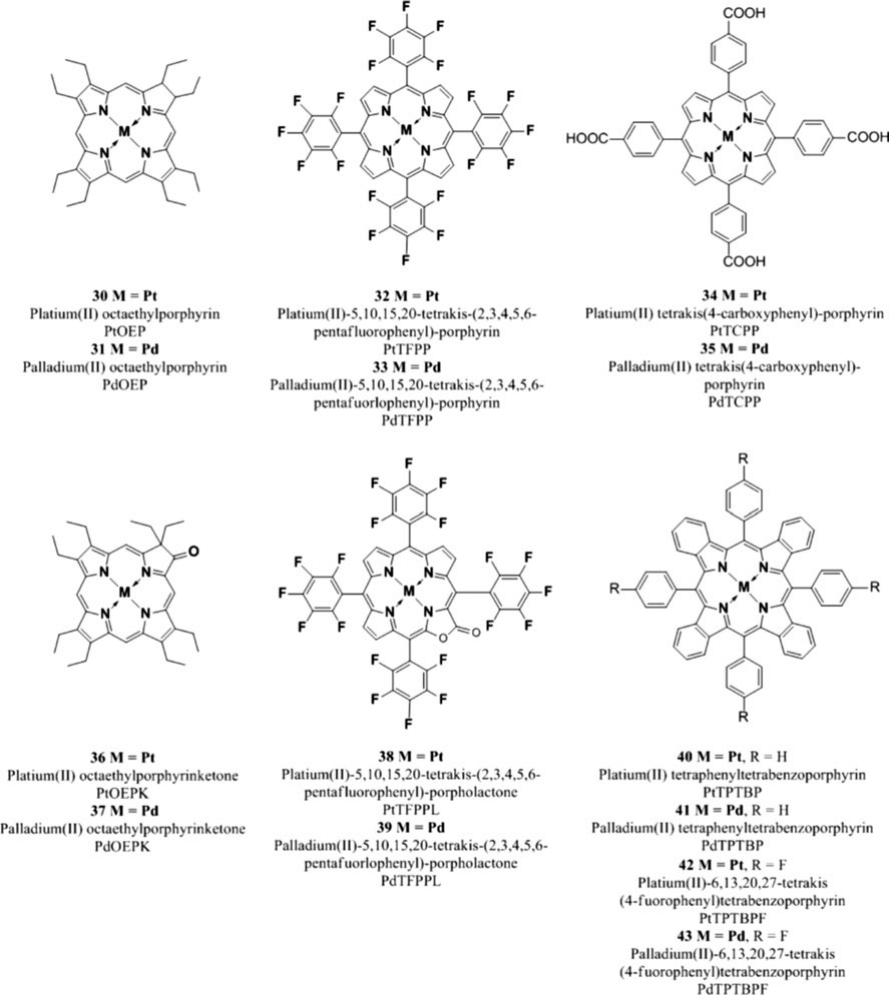
Chemical structures of Pt(II) and Pd(II) porphyrin complexes

The complexes possess excellent brightness upon excitation both in the blue and red region due to very high molar absorption coefficients and quantum yields. For example, molar absorption coefficients for the platinum(II) and palladium(II) complexes with tetraphenyltetrabenzoporphyrin PtTPTBP **40** and PdTPTBP **41** (Fig. 8), respectively, exceed 200,000 M^−1^ cm^−1^ for the blue region and 130,000 M^−1^ cm^−1^for the red region, and the emission quantum yields are about 0.50 and 0.20, respectively. The Pd(II) complexes with tetraphenyltetrabenzoporphyrin are water insoluble and not cell penetrating, but once incorporated into polystyrene nanoparticle, functionalized with polyethylene glycol (PEG) and an antibody, they were successfully used for in vivo imaging [170].

PtTPTBPF **42** and PdTPTBPF **43** (Fig. 8) are obtained by substitution of the hydrogen to fluorine in the *meso*-phenyl rings, which slightly improves the luminescence quantum yield of both Pt(II) and Pd(II) complexes (Table 3) and enhances their photostability [27].

Solubility of an indicator in polymeric matrices is an important parameter for its application in optical sensors; planar π-extended porphyrins were shown to aggregate readily, but substituents in the *meso*-position of the porphyrin ring significantly improve their solubility. For example, *meso*-substituted benzoporphyrins as **40**, **41**, **42**, and **43** show a low tendency to aggregate in nonpolar polymers such as polystyrene [28].

In general, when dissolved in polystyrene, the Pt(II) *meso*-substituted benzoporphyrins are ideally suitable for measurements from 0 to 100 % air saturation while the analog Pd(II) dyes, as they show a significantly higher sensitivity to oxygen with decay times in the order of 300 μs, are more adequate for trace oxygen sensing because their luminescence is almost completely quenched at air saturation. However, the Pd(II) complexes could be used to measure oxygen up to air saturation when incorporated into polymers with lower oxygen permeability such as poly(styrene-*co*-acrylonitrile).

**Fig. 9:**
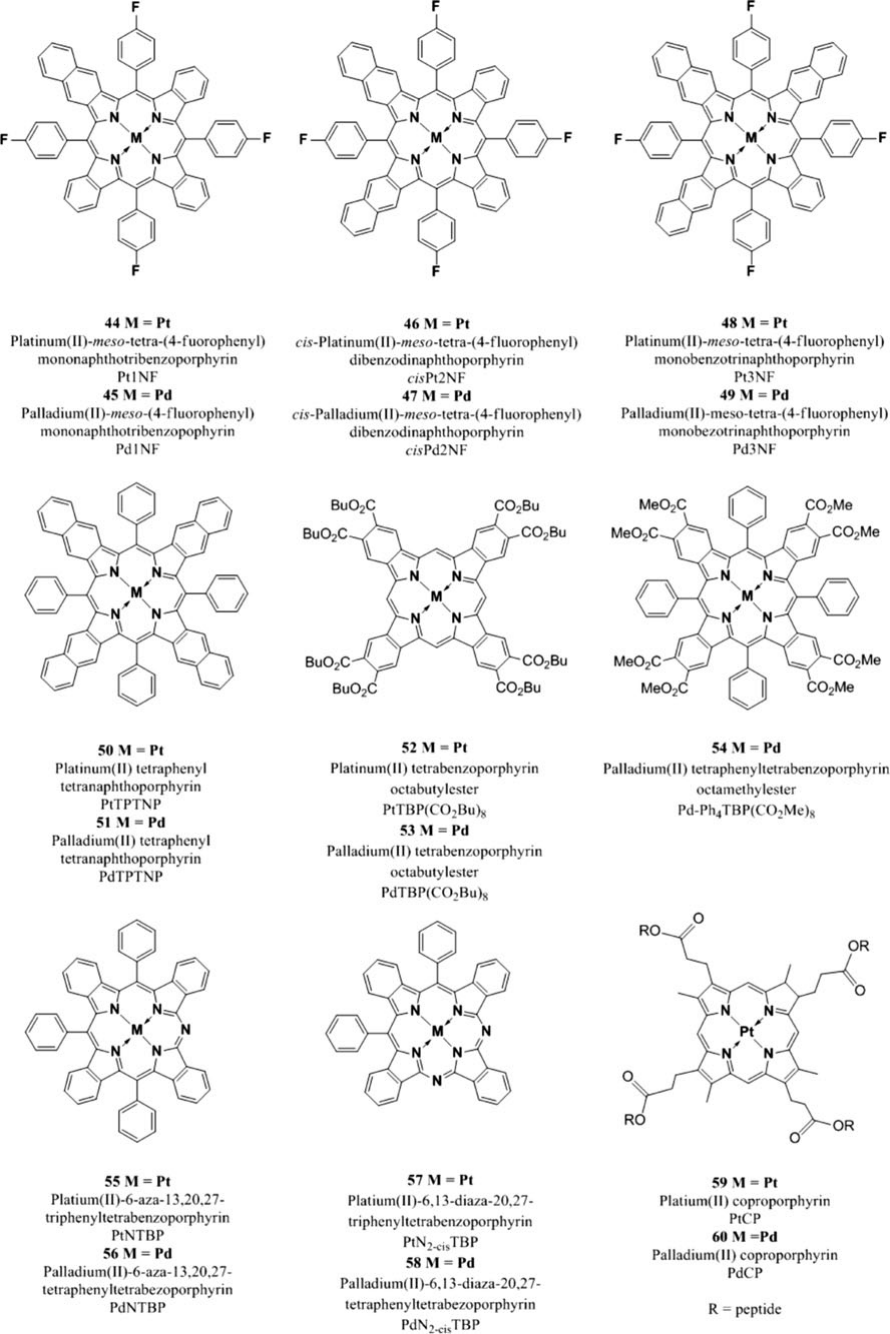
Chemical structures of Pt(II) and Pd(II) porphyrin complexes

NIR indicators efficiently absorb light at wavelengths higher than 600 nm; therefore, they have the potential to be used in the so called “smart tattoos”. These are sensors that are implanted into subcutaneous tissues and can be exploited for continuous noninvasive monitoring of vital analytes like oxygen or glucose [28]. Further, bathochromic shifts of the absorption and emission can improve the performance in such applications even more. This can be realized by further extension of the π-system as for tetranaphthoporphyrins TNP (Fig. 9). Compared to the benzoporphyrins, the absorption (for the Q bands) and emission of these dyes shift bathocromically by about 80 nm (Table 3). Unfortunately, this is accompanied by the decrease in the phosphorescence quantum yields (*Φ*_*P*_ = 0.22 for PtTPTNP **50** and *Φ*_*P*_ = 0.079 for PdTPTNP **51**) [71, 73]. Even more importantly, the photostability of these dyes decreases dramatically [148] in comparison with the corresponding tetrabenzoporphyrins; which can compromise potential application in oxygen sensing, organic light-emitting diodes (OLEDs) [207], and photovoltaics [52].

Lower solubility of the *meso*-tetraphenyltetranaphthoporphyrins is another drawback, but other more soluble derivatives are known and will be discussed below. It should be mentioned here that a synthetic way to render halogenated naphthoporphyrins significantly more photostable and better soluble have been recently described [70]. However, the multistep synthesis of precursors and the porphyrins is not unchallenging.

Hybrid benzo- and naphthoporphyrin complexes of platinum(II) and palladium(II) with tunable spectral properties were reported by Niedermair et al. [172] The photophysical properties of the platinum(II)-*meso*-tetra-(4-fluorophenyl)-mononaphthotribezoporphyrin (Pt1NF **44**), the *cis*-platinum(II)-*meso*-tetra-(4-fluorophenyl)dibenzodinaphthoporphyrin (*cis*-Pt2NF **46**) and the platinum(II)-*meso*-tetra-(4-fluorophenyl)monobenzotrinaphthoporphyrin (Pt3NF **48**) and the corresponding palladium(II) complexes (Pd1NF **45**, cis-Pd2NF **47**, Pd3NF **49**; Fig. 9) are reported in Table 3. These complexes absorb intensively in the near-infrared region between 628 and 691 nm (the higher the number of the naphtha moieties annealed to the porphyrin system, the higher the bathochromic shift) and emit at room temperature between 815 and 882 nm. The phosphorescence quantum yields decrease with the number of naphtha moieties and they range from 0.53 to 0.25 for the Pt(II) complexes and from 0.18 to 0.07 for the Pd(II) complexes. The luminescence decay times follow the same trend decreasing from 44 to 21 μs for the Pt(II) complexes and from 203 to 106 μs for the analog Pd(II) complexes; therefore, it can be stated that the sensitivity to oxygen is affected and gradually reduces upon substitution. It has been reported that the decrease in photostability of these indicators correlates well with the number of naphtho moieties annealed to the porphyrin system. Thanks to their tunable properties, the described chromophores are good candidates for different multiplexing applications as for example the simultaneous measurement of glucose and oxygen in enzymatic sensors [172].

Platinum(II) and palladium(II) tetrabenzoporphyrin-octabutylester (PtTBP(CO_2_Bu)_8_ **52**, PdTBP(CO_2_Bu)_8_ **53**; Fig. 9) and the palladium(II) tetraphenyltetrabenzo-porphyrin-octamethylester ((Pd-Ph_4_TBP)CO_2_Me)_8_ **54** (Fig. 9) represents another variation in the class of π-extended porphyrins which were synthesized and characterized by Vinogradov and co-workers [73].

The absorption bands of the *meso*-unsubstituted tetrabenzoporphyrins (**52** and **53**) are blue shifted by about 10–30 nm when compared to the corresponding bands of the TPTBP analog (**40–41**; Table 3) while the emission decay times and quantum yields are comparable.

Another class of red excitable dyes for optical oxygen sensing is the platinum(II) and palladium(II) complexes with azatetrabenzoporphyrins recently reported by Borisov et al. [31]. These compounds can be viewed as hybrids between tetrabenzoporphyrins and phthalocyanines and combine the advantages of both. Pt(II) and Pd(II) phthalocyanines are characterized by extremely good chemical, thermal, and photostability but their phosphorescence quantum yields are very poor (*Φ*_*P*_ < 0.01) [190] which makes them hardly feasible for optical sensing or imaging of oxygen. The photophysical properties of platinum(II) 6-aza-13-20-27-triphenyltetrabenzoporphyrin (PtNTBP **55**), platinum(II) 6-aza-13-20-27-triphenyltetrabenzoporphyrin (PtN_2-*cis*_TBP **57**) and the corresponding palladium(II) analog (PdNTBP **56**, PdN_2-*cis*_TBP **58**) are reported in Table 3. When compared to the respective TPTBP complexes, they show a hypsochromic shift of the Soret band and a bathochromic shift of the Q bands. In general, the decay times and the emission quantum yields of complexes **55–58** are lower when compared to the ones of the respective *meso*-tetraphenyltetrabenzoporphyrins; but despite this, the brightness of such indicators is quite adequate for practical applications and the lower sensitivity can be exploited in application where the oxygen partial pressure is higher than 21 kPa (e.g., in photosynthetic systems). Importantly, the aza-modified complexes were shown to possess a much higher photostability than the corresponding TPTBP complexes.

Porphyrin conjugates and dendrimers represent another group of oxygen probes and have been reported both for non-extended and π-extended porphyrins. Usually, the aim of the synthetic modifications is to provide additional functionality (water solubility, partial shielding from oxygen and other species, cell penetration, recognition of specific cells or cellular components, etc.).

Recently, Papkovsky and co-workers [57] reported on a panel of phosphorescent oligoarginine conjugates of tetracaboxylic Pt(II) and Pd(II)-coproporphyrin I (PtCP **59** and PdCP **60**; Fig. 9) mono or tetra-substituted, which were successfully used for sensing intracellular oxygen thanks to their ability to penetrate cells. The authors showed that all the Pt(II)-tetra-substituted conjugates displayed absorption spectra that were similar to those of the free PtCP; on the other hand, it was reported that the conjugation with peptides had a significant decreasing effect on the emission quantum yield (see Supporting Information of [57]) while all the PtCP conjugates displayed very similar phosphorescence lifetimes (20–23 μs in air saturated and ~80 μs in deoxygenated media).

The corresponding PdCP conjugates, as expected, showed significantly longer unquenched phosphorescence lifetimes (~1 ms) and therefore are better suited to measure oxygen at very low concentrations.

**Fig. 10:**
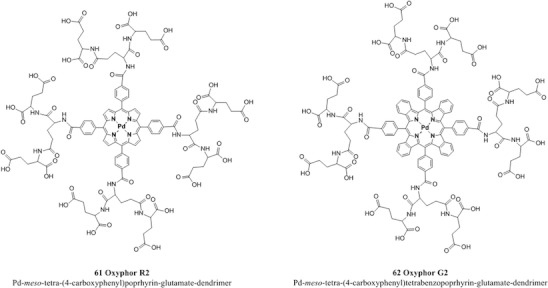
Chemical structure of Oxyphor R2 and Oxyphor G2

Metalloporphyrins are generally hydrophobic and therefore insoluble in water or in physiological fluids; they therefore need to be modified by addition of “molecular coats” in order to enhance their interactions with the environment when used, for example, to measure the oxygenation of tumors in vivo [63]. Oxyphors R2 **61** and G2 **62** (Fig. 10) have been constructed by modification of Pd(II)-*meso*-tetra (4-carboxyphenyl)-porphyrin and Pd(II)-*meso*-tetra (4-carboxyphenyl)-tetrabenzoporphyrin, respectively, and are the second generation of polyglutamic dendrimers. The outer layers of the dendrimers have 16 carboxylate groups which are responsible for both their solubility in biological fluids and their inability to pass through biological membranes. The photophysical properties of the two dendrimers are shown in Table 3 and it can be highlighted that Oxyphor **G2** could be the indicator of choice when measuring tissue oxygenation that require light penetration into the depths of tissue since it has an absorption band near 632 nm.

**Fig. 11:**
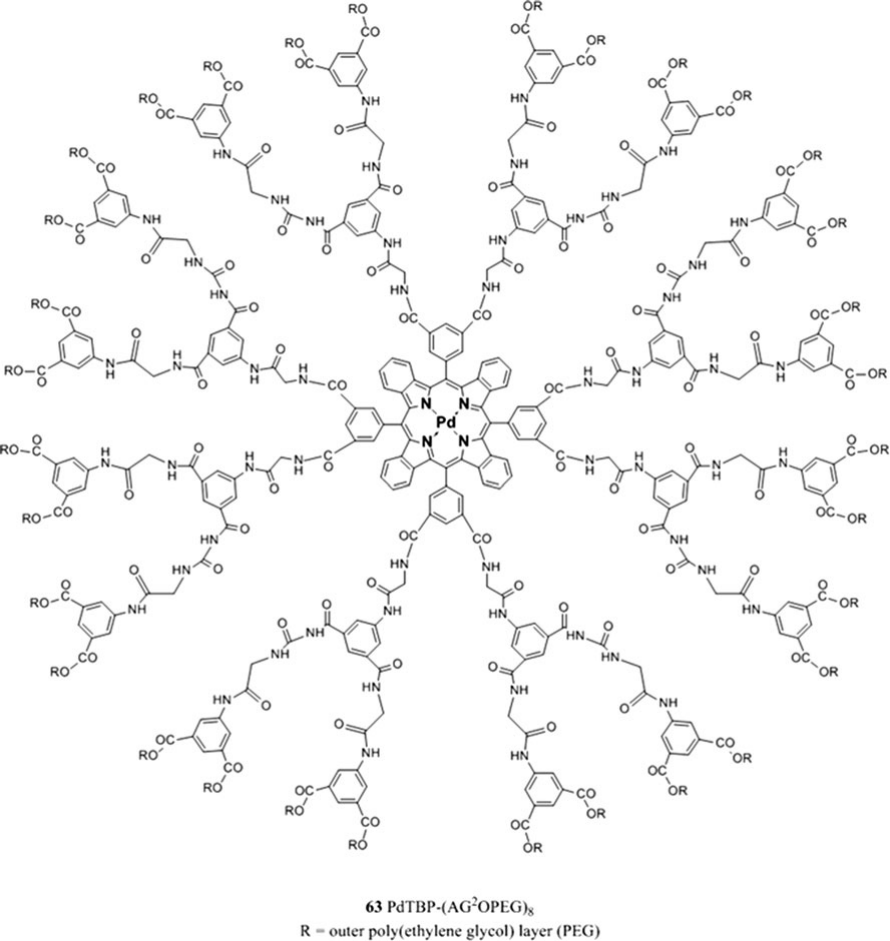
Schematic structure of PEG modified PdTBP-dendrimer

The same phosphor **G2** was incorporated into nanoparticles (PEBBLES) modified with peptides to be used for intracellular oxygen measurements [135]. Metalloporphyrins of palladium(II) and platinum(II) have been also encapsulated into poly(arylglycine) dendrimers modified with poly(ethyleneglycol) residues in order to enhance the dye’s solubility, diminish its toxicity and help preventing its interaction with the biological environment when used in vivo [130]. The dendrimer folds in aqueous environments and creates a diffusion barrier for oxygen which can be exploited to regulate the sensitivity and the dynamic range of the probe. The photophysical properties of such a probe PdTBP-(AG^2^OPEG)_8_ **63** (Fig. 11) are reported in Table 3. Several other water-soluble dendrimers were reported recently by the same group [66]. Vinogradov and co-workers have also reported dendrimeric oxygen probes bearing coumarin antennas along with a porphyrin core [32, 74]. These probes were found useful for application in two-photon microscopic imaging of oxygen distribution in tissues due to the fact that the coumarin antennas increased the efficiency of the two-photon excitation compared to the dendrimers solely based on the benzoporphyrin complexes.

It should be mentioned here that lipophilic porphyrins encapsulated into water-dispersible nanoparticles represent an alternative to dendrimeric oxygen probes and often perform similarly despite their significantly larger dimensions (typically tens of nanometer). The polymer of the particle provides necessary protection for the indicator dye from the environment and helps to tune oxygen sensitivity. The functional groups on the surface of the particles not only render them water-dispersible but also can enable, e.g., cell penetration [69].

**Fig. 12:**
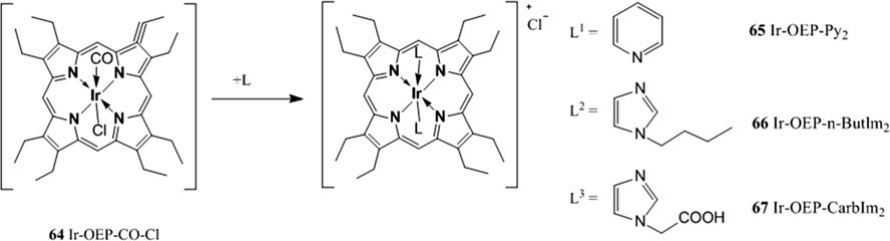
Chemical structures of Ir(III) porphyrins complexes

Recently, Koren et al. [115] reported the synthesis and characterization of new Ir(III) porphyrin complexes. In contrast to the square planar Pt(II) and Pd(II) porphyrins, the new hexa-coordinating complexes showed quite interesting photophysical properties which could be tuned by modification of the axial ligands, modifications that demonstrated to have an influence also on the solubility of the complexes. Three different indicators were synthesized from the same parent complex (Ir-OEP-CO-Cl **64** - Fig. 12) by ligand exchange and their photophysical properties are reported in Table 3 together with the ones of the parent complex **64**. The positively charged complexes Ir-OEP-Py_2_ **65**, Ir-OEP-*n*-butIm_2_ **66**, and Ir-OEP-CarbIm_2_ **67** with two similar axial ligands show similar properties, in contrast to the neutral Ir-OEP-CO-Cl; they all have relatively high phosphorescence quantum yields (up to 21 %) but shorter decay times. The absorption and emission spectra of **65–67** are bathochromically shifted compared to the Pt(II) complexes and therefore they can be excited with visible light directly in the Soret band (Fig. 12).

The axial ligands can be used not only to modify the solubility of the complexes but also to introduce functional groups to enable, for example, covalent coupling. For example, very recently, Koren et al. [116] directly attached short peptides to the Ir(III) complex via a hystidine unit at the *N*-terminus of the peptide and used the new probes for oxygen imaging in mammalian cells.

Very recently, an oxygen probe based on a copolymer of a Pt(II) porphyrin and 9,9-dioctylfluorene has been reported [246]. The conjugated polymer combines the properties of a luminescent indicator and a polymeric matrix and, more importantly, possesses dual emission under UV/violet excitation which originates from the fluorene (oxygen-independent fluorescence) and Pt(II) porphyrin (oxygen-dependent phosphorescence). Thus, ratiometric sensing and imaging becomes possible; the authors also demonstrated that the ratio of the emission intensities can be adjusted by varying the ration of fluorene/Pt(II) porphyrin units.

4. Cyclometallated complexes

Another class of indicators which have been exploited for oxygen sensing is represented by the cyclometallated complexes of Ir(III) and Pt(II). The last decade has seen an enormous progress in the field of luminescent cyclometallated complexes which was driven by the development of OLED technology. Some of these dyes were also applied as oxygen indicators.

In general, thesee complexes show high luminescence quantum yields, have large Stokes’ shift and are photostable. However, their luminescence decay times are in order of several microseconds; therefore, usually much shorter than the one of metalloporphyrins, also their absorption in the visible region is not efficient (*ε* rarely exceeds 10,000 M^−1^ cm^−1^).

The *tris*(2-phenylpyridine) iridium(III) [Ir(ppy)_3_] **68** displays a very strong green luminescence with high quantum yield (0.90) and relatively long lifetime (1.5 μs; Table 4). The molar absorption coefficient in the visible region is comparable to other cyclometallated compound and is rather low (<10,000 M^−1^ cm^−1^) [126]. This indicator has been used as oxygen sensor immobilized in different matrices such as: poly(styrene-*co*-trifluoroethylmetacrylate (TFEM)) in which it showed quite good photostability [7], Ormosil where it was used as a transducer for the detection of uric acid [201], and poly(dimethylsiloxane) which was functionalized with the Ir(III) complex and blended with polystyrene [117]. Very recently, complex **68** has been modified to be suitable for imaging living cells; to enable cellular uptake the phenylpyridine ligands were functionalized with three different amino acids (lysine, alanine, and glycine) [211]. The modified complexes were shown to possess relatively high cellular uptake but, compared to **68**, their quantum yields were only moderate (< 0.2).

**Table 4:** Photophysical properties of cyclometallated complexes of Ir(III) and Pt(II)

Dye	*λ*_max_^abs^ (nm) (ε × 10^3^ M^−1^ cm^−1^)	*λ*_max_^em^ (nm)	Medium	*Φ*	*τ*	References
**68** Ir(ppy)_3_	376	512	THF	0.90	1.5 μs	[7]
**69** Ir(ppy-NPh_2_)_3_	405	527	2-MeTHF	0.70	4.3 μs	[149]
**70** Ir(btpy)_3_	292 (67.6)/366 (20.4)/408 (21.8)	596	CHCl_3_	–	8.6 μs^a^	[77]
		645				
**71** N-948^b^	494	665	CH_2_Cl_2_	0.58^b^	102 μs^c^	[157]
**72** Ir(C_N_)_2_(acac)	450 (57.1)/421 (86.9)	545	CHCl_3_	0.53	8.5 μs	[24]
**73** Ir(C_O_)_2_(acac)	467 (53.7)/443 (47)	552	CHCl_3_	0.34	10.7 μs	[24]
**74** Ir(C_S-Me_)_2_(acac)	471 (75.4)/441 (77.8)	564	CHCl_3_	0.44	11.3 μs	[24]
**75** Ir(C_S_)_2_(acac)	472 (92.8)/444 (86.8)	563	CHCl_3_	0.54	11.3 μs	[24]
**76** (C_N_)_2_Ir(μ-Cl)_2_Ir(C_N_)_2_	463 (87.6)/433 (133.6)	568	CHCl_3_	0.30	9.7 μs	[24]
**77** (C_S_)_2_Ir(μ-Cl)_2_Ir(C_S_)_2_	482 (136.5)/457 (136.9)	587	CHCl_3_	0.21	13.1 μs	[24]
**78** Ir(C6)_2_(vacac)	445^d^ (77.9)/474^d^ (86.9)	568	Toluene	0.22	6.0 μs	[55]
**80** Pt(thpy)_2_	470 (2)	~590	Acetonitrile	0.36	4.8 μs	[59]
**81** C^N_1_Pt(acac)	459 (13.0)	502	Toluene	0.95^e^ 0.22^e^	0.06 ns 1.53 μs	[143]
		535				
		683				
		753				
**82** C^N_2_Pt(acac)	474 (21.7)	521	Toluene	3.03^e^	0.14 ns	[143]
		556				
		692		0.57^e^	3.74 μs	
		762				
**83** C^N_3_Pt(acac)	243 (52.0) 390 (33.7)	455	CH_2_Cl_2_	1.1^e^	3.2 ns	[244]
		638			6.6 μs	
**84** C^N_4_Pt(acac)	357 (1.73) 400 (0.41)	386 538	CH_2_Cl_2_	18.2^e^	25 ns	[244]
					25.5 μs	
**85** C^N_5_Pt(acac)	292 (2.76) 396 (0.51)	536	CH_2_Cl_2_	32.3^e^	5.4 μs	[244]
**86** C^N_6_Pt(acac)	234 (38.1) 339 (25.1)	575	CH_2_Cl_2_	9.1^e^	15.8 μs	[244]
**87** C^N_7_Pt(acac)	335 (2.68) 400 (0.63)	560	CH_2_Cl_2_	4.5^e^	0.86 μs	[244]
**88** dppe-Pt2P	344^d^ (12.4)/496^d^ (3.2)	677	DMSO	0.002	0.2 ns	[220]
		732		0.01	8.66 μs	

^a^Measured at 50 mbar air pressure and 1 °C

^b^ *N*-948 = Ir(2-phenylpyridine)_2_(4,4′-*bis*(2-(4-*N*,*N*-methylhexylaminophenyl)ethenyl)-2,2′-bipyridine)

^c^When incorporated in polystyrene (PS)

^d^Measured in dichloromethane

^e^Obtained in toluene with Ru(byp)_2_(phen)[PF_6_]_2_ as the standard

A substitution of the ppy ligands in complex **68** with ppy-NPh_2_ leads to the formation of a new cyclometalated indicator: [Ir(ppy-NPh_2_)_3_] **69** which has the advantage of being less sensitive to self-quenching and is even better soluble in organic solvents and organic polymers [149]. Complex **69** has a high phosphorescence quantum yield (~70 %), lifetime in the order of several microseconds (Table 4), can be excited at 405 nm and therefore is compatible with low-cost LED as excitation source and its excitation and emission spectra are well shifted. A third complex possessing the same skeleton as **68** has been used as red-emitting dye: the *tris*{2-(benzo[b]thiophene-2-yl)pyridinato-C^3^,N}iridium(III) **70**; which is characterized by large Stokes’ shift and a lifetime of 8.6 μs that is suitable for lifetime imaging application. However, similarly to other cyclometallated complexes the absorption is very efficient only in the UV region (ε as high as 60,000 M^−1^ cm^−1^ at 292 nm Table 4). Ir(btpy)_3_ has been used, along with a green-emitting iridium dye, in a dual pressure and temperature sensitive paint [77]. The barometric dye **70** was incorporated in a cellulose acetate butyrate film and the cross-sensitivity to temperature was efficiently corrected by simultaneous optical determination of the temperature.

The [Ir(2-phenylpyridine)2(4,4′-*bis*(2-(4-N,N-methylhexylaminophenyl)ethyl)-2,2′-bipyridine)Cl] **71** which for convenience was given the name N-948 possesses luminescent emission at a wavelength higher than 650 nm (665 nm; Table 4). N-948 was shown to have quite good quantum yields (*Φ*_*P*_ > 0.50), extraordinary long decay time (102 μs when incorporated in polystyrene) and high lipophilicity which increase its retention in rather apolar polymeric films. N-948 was incorporated in both polystyrene and a nanostructured metal oxide matrix which increased its photostability and resistance to heat and γ-irradiation during sterilization.

**Fig. 13:**
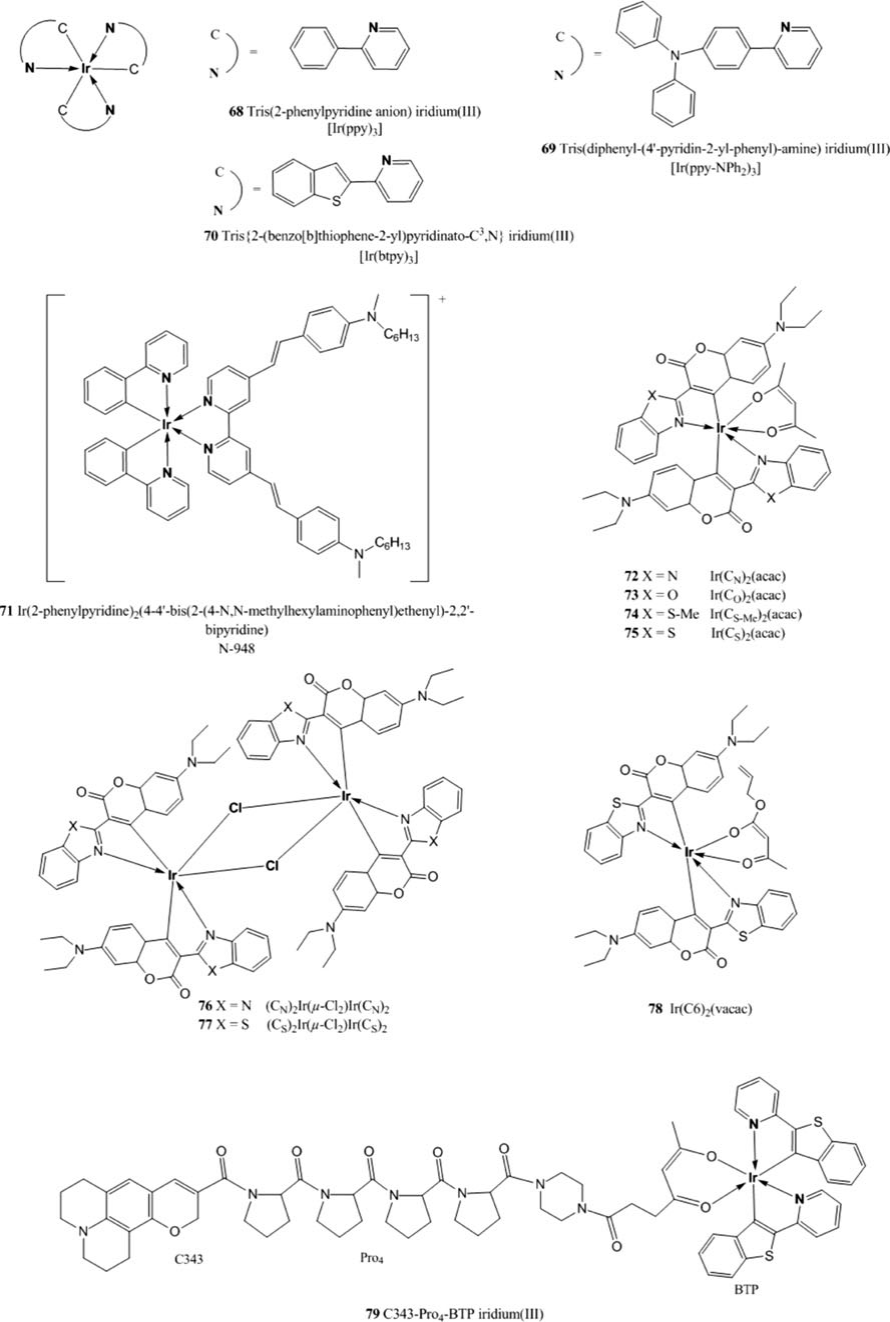
Chemical structures of selected Ir(III) cyclometallated complexes

As was mentioned above, rather low molar absorption coefficients of the cyclometallated complexes represent a serious disadvantage for their application as oxygen indicators. Cyclometalated iridium(III) complexes with coumarins overcome this drawback [24, 126]. Such indicators are characterized by efficient visible absorption in the blue part of the spectrum (ε as high as 90,000 M^−1^ cm^−1^; Table 4) and very strong phosphorescence (*Φ*_*P*_ ~0.50) which results in an exceptionally high brightness and makes them attractive for application in thin films to monitor fast processes and different types of nano- and microparticles. The photophysical properties of cyclometalated Ir(III) coumarin complexes of general formula Ir(C_X_)_2_(acac) where *X* = *N* (**72**), O (**73**), S-Me (**74**) and S (**75**) are reported in Table 4 and their chemical structures shown in Fig. 13. The coumarin substituent represents a rather flexible system in respect to fine-tuning of spectral properties of the complexes, which depend on the *X* substituent of the coumarin: the bathochromic shift increases when *X* = *N* > O > S [24]. As already mentioned, these indicators show high luminescence brightness (ε × *Φ*_*P*_ exceeds 50,000 for Ir(C_S_)_2_(acac) and it is as high as 46,000 for Ir(C_N_)_2_(acac)). It should be also mentioned that all the complexes **72–75** can be efficiently excited with 425, 435, and 450 nm LEDs and possess relatively sharp excitation and emission bands therefore are suitable for multi-analyte sensing. The phosphorescent decay times for this class of ultrabright indicators are of ~10 μs for all the complexes which insures good sensitivity in polystyrene-based materials [24].

The dimeric coumarin complexes of general formula (C_X_)_2_Ir(*μ*-Cl)_2_Ir(C_X_)_2_ were also investigated (**76–77**, Fig. 13). However, the emission quantum yields are lower than the respective monomeric complexes (Table 4). The absorption and emission maxima of the dimeric complexes shift bathochromically and in general the shift in the emission maxima is more pronounced.

De Rosa et al. presented a derivative of a coumarin complex, Ir(C6)_2_(vacac) **78** (Fig. 13) which was covalently bound to a polymeric support (silicone rubber) to eliminate dye aggregation. Unfortunately, the indicator in this case exhibited lower brightness in respect to the unbounded derivative. Nevertheless, complex **78** displayed good quantum yields and luminescence lifetime (Table 4) and was suggested as a good candidate for PSP studies [55].

Very recently, Yoshihara et al. [254] presented a novel intrinsic ratiometric molecular probe, the C343-Pro_4_-BTP **79** (Fig. 13) which consists of an oxygen-sensitive phosphor (Ir(III)BTP, BTP = *bis*(2-(2′-benzothienyl)-pyridinato-N,C^3^)) connected to an oxygen-insensitive fluorophore (coumarin C343) by a tetraproline linker. Ideally, this probe was designed to measure local oxygen levels in living cells and tissue but currently cellular uptake is too low to perform quantitative analysis but the proline linker can be modified to enhance the delivery to cells.

As previously mentioned, also Pt(II) cyclometalated complexes have been used as indicators for oxygen sensors and a selection is reported here. *Cis*-*bis*[2-(2′-thienyl)pyridine]platinum(II), Pt(thpy)_2_ **80** was selected as a luminophore for sensing O_2_ concentration in seawater [59]. This indicator can be easily excited with blue LED as it absorbs light up to 500 nm, it possesses high luminescence quantum yield (*Φ*_*P*_ = 0.36) and an excited-state lifetime of 4.8 μs. The non-ionic character makes it basically insoluble in water and therefore it is not susceptible to leaching in presence of water. Similarly to the analogous Ir(III) complexes, cyclometallated Pt(II) indicators possess moderate to low absorption in the visible part of the spectrum. It should be mentioned here that as recently demonstrated by Hanson et al. [90], the visible absorption of the cyclometallated complexes can be enhanced by introducing a highly absorbing ligand in addition to the cyclometallated one. Although this approach has not yet been applied in oxygen sensors, it represents an elegant solution.

**Fig. 14:**
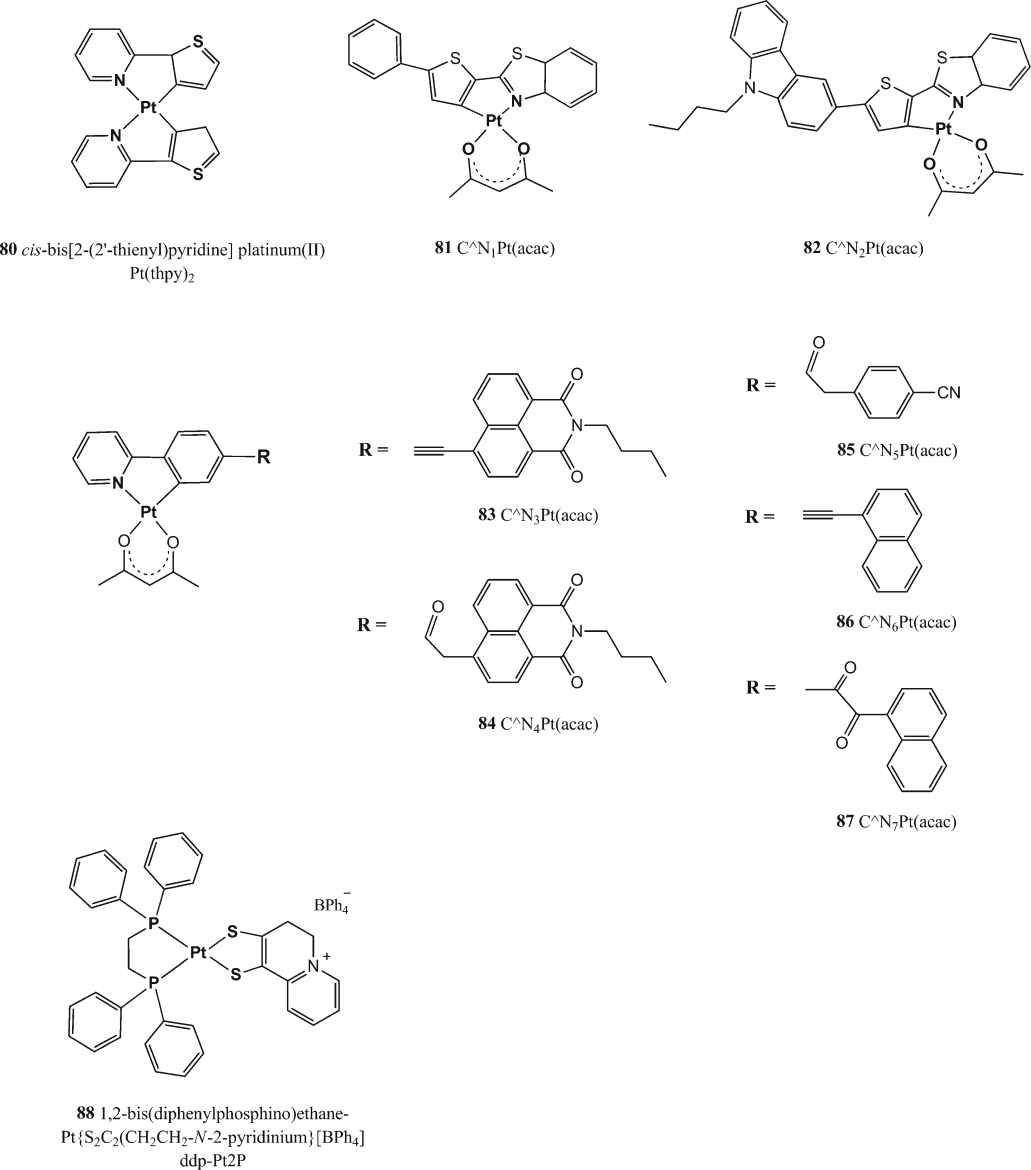
Chemical structures of selected Pt(II) cyclometallated complexes

As mentioned earlier in this review, it would be highly desirable to design a ratiometric sensor for imaging which uses a single molecule. Only few examples of such indicators were found in the literature, which is understandable since such probes are not easy to design, it is in fact difficult to predict if a molecule will show both fluorescent and phosphorescent emissions and in comparable quantum yields. Some examples of such indicators were recently reported by Zhao et al. [143, 244]. They entailed cyclometalated Pt(II) complexes of general formula C^NPt(acac), some of which show dual fluorescence and phosphorescence emission. The structures of complex **81–87** are shown in Fig. 14 and their photophysical properties, which mainly depend on the C^N cyclometallating ligand, are reported in Table 4. Some of the complexes show enhanced absorption in the visible region due to the presence of naphthalimide chromophore and **81- 84** presented well-separated dual emission bands.

C^N_1_Pt(acac) **81** gives fluorescence emission bands at between 500 and 650 nm with a quantum yield of 0.95 and a lifetime of 0.06 ns, this bands are basically unaffected by the presence of O_2_. On the other hand, the phosphorescence emission bands at 650–850 nm with quantum yields of 0.22 and lifetime of 1.53 μs are quite sensitive toward variation of oxygen concentration. Similar behavior was shown by C^N_2_Pt(acac) **82** which was found to be more sensitive due to its longer phosphorescence lifetime (3.75 μs).

C^N_3_Pt(acac) **83** is the first of a series of (ppy)Pt(acac) complexes which differ from each other by the R substitution in the ppy ligand (Fig. 14). Complex **83** is the only one showing deep NIR emission (638 nm) with and emission lifetime of 6.6 μs. C^N_4_Pt(acac) **84**, which has the same subunit as **83** but attached to the ppy ligand via a methylene-keto group, has a blue-shifted emission at 538 nm and a longer luminescent lifetime (25.5 μs). Minor emission bands for both **83** and **84** could be exploited for ratiometric dual emission measurements.

C^N_5_Pt(acac) **85**, C^N_6_Pt(acac) **86**, C^N_7_Pt(acac) **87** emit roughly at the same wavelength range (530–570 nm) but show different sensitivities towards oxygen quenching as they show quite different decay times, with C^N_6_Pt(acac) **86** having the longer one (15.8 μs).

Another complex belonging to the same class of dual emitters for oxygen ratiometric measurements is the 1,2-*bis*(diphenylphosphino)ethane-Pt{S_2_C_2_(CH_2_CH_2_-*N*-2-pyridinium)}[BPh_4_], ddpePtP2 **88** [220]. Excitation of **89** leads to a dual emission: a fluorescent one at 677 nm with a quantum yield of 0.002 and lifetime of 0.2 ns and a phosphorescent one at 732 nm with a quantum yield of 0.01 and lifetime of 8.66 μs. The triplet intensity decreases approximately 2.5-fold on changing from nitrogen to oxygen while the singlet intensity is basically unaffected; this feature can be therefore used for ratiometric measurement [118]. A complication arises from a significant overlap between the peaks of excitation and emission spectra; to overcome this problem the authors utilized a method that switches between two frequencies of excitation modulation and excludes any overlapping effects. Unfortunately both emissions are rather weak, which is a very serious drawback.

5. Complexes with rarely used central atoms

**Fig. 15:**
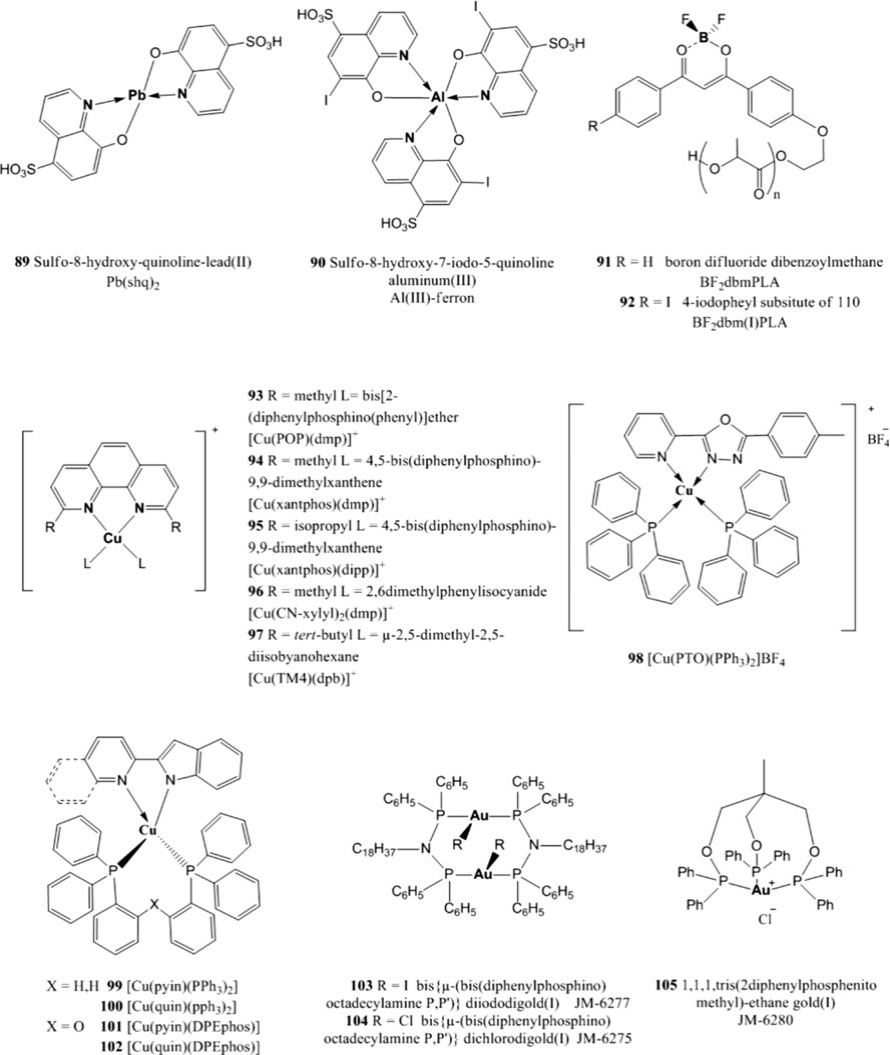
Chemical structures of selected Al(III), Pb(II), Cu(I), Au(III), and boron complexes

**Fig. 16:**
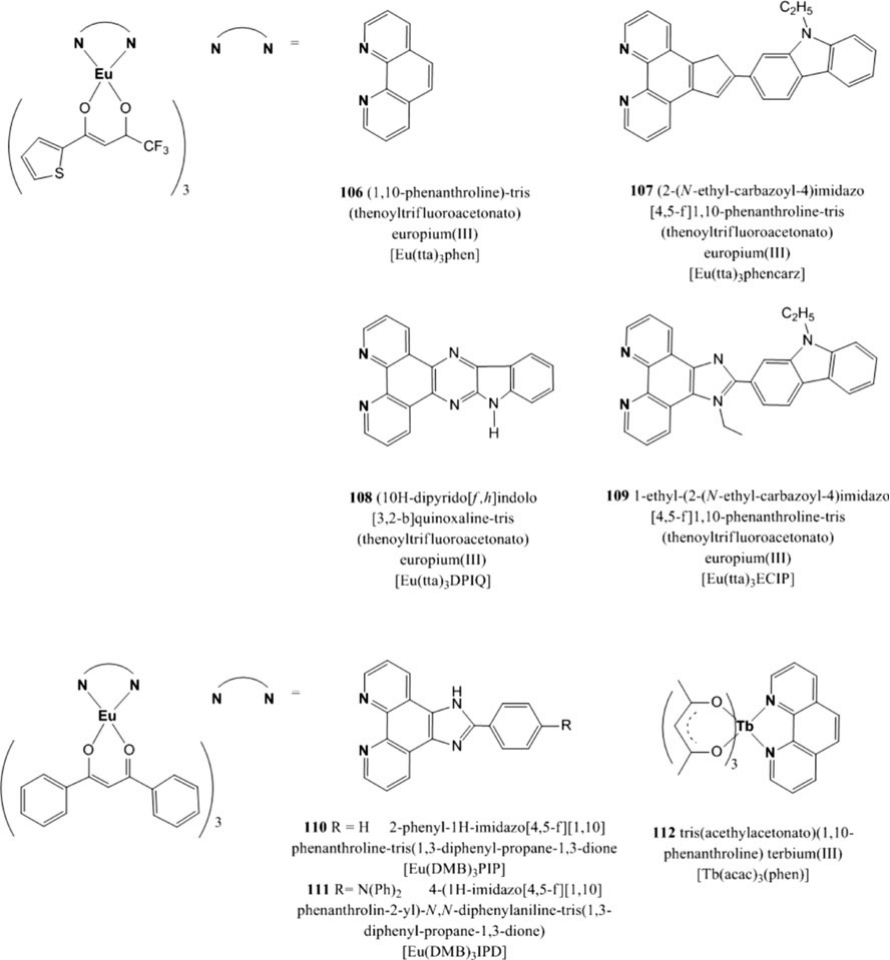
Chemical structures of selected Eu(III) and Tb(III) complexes

Under this section, the reader can find those indicators for optical oxygen sensing which possess a central atom that has been used less commonly (Figs. 15 and 16). The primary motivation behind the research of different materials is to provide an alternative to rather expensive platinum group metals as central atoms which can be of relevance for certain applications (e.g., food packaging where the price of the sensing material is one of the most important parameters to be considered).

Similar to the Ru(II) polypyridyl complexes, the luminescence of Pb(II) complexes is dominated by the metal-to-ligand charge transfer process that involves the promotion of an electron from a metal *d* orbit to a ligand π^*^ orbit.

When the lead(II) complex of 8-hydroxy-5-quinolinesulfonic acid (**89**) is retained on the surface of anion-exchange resin beads, it exhibits room temperature phosphorescence in suspensions of both aqueous and organic solutions [1]. This complex is characterized by long emission lifetime (Table 5) and very good photostability.

**Table 5:** Photophysical properties of complexes with rarely used central atoms

Dye	*λ*_max_^abs^ (nm) (*ε* × 10^3^ M^−1^ cm^−1^)	*λ*_max_^em^ (nm)	Medium	*Φ*	*τ*	References
**89** Pb(shq)_2_	450	638	Toluene	–	0.16 ms	[1]
**90** Al(III)-ferron	390	600	Water	–	460 μs^a^	[144]
**91** BF_2_dbmPLA	396^b^ (50.1)	440 509	Thin film	0.89^c^	1.2 ns	[256]
					170 ms	
**92** BF_2_dbm(I)PLA	406^b^ (33)	485 535	Powder	0.41^c^	0.37 ns	[258]
					4.06 ms	
**93** [Cu(POP)(dmp)]tfbp	–	517	Solid state	0.88^d^	26.0 μs	[205]
**94** [Cu(xantphos)(dmp)] tfbp	–	540	Solid state	0.66^d^	30.2 μs	[205]
**95** [Cu(xantphos)(dipp)] tfbp	–	513	Solid state	0.95^d^	38.5 μs	[205]
**96** [Cu(CN-xylyl)_2_ (dmp)] tfbp	~270^e^	520	Solid state	0.228^d^	309 μs	[206]
**97** [Cu(TM4)(dmp)]_2_ (tfbp)_2_	~270^e^	530	Solid state	0.226^d^	1.2 ms	[206]
**98** [Cu(PTO)(PPh_3_)_2_]BF_4_	220/340	515	CH_2_Cl_2_	–	64.4 μs^b^	[88]
**99** [Cu(pyin)(PPh_3_)_2_]	232^f^ (40.5) 324^f^ (15.5)	562 474	PMMA film	0.08	1.17 μs	[145]
					4.66 ns	
**100** [Cu(quin)(PPh_3_)_2_]	231^f^ (53.1) 354^f^ (15.6)	608 550	PMMA film	0.042	1.32 μs	[145]
					5.04 ns	
**101** [Cu(pyin)(DPEphos)]	231^f^ (32.4) 324^f^ (14.9)	566 475	PMMA film	0.05	1.20 μs	[145]
					4.78 ns	
**102** [Cu(quin)(DPEphos)]	231^f^ (38.1) 354^f^ (13.9)	610 550	PMMA film	0.044	1.59 μs	[145]
					4.89 ns	
**103** JM-6277	418^g^	530	PS	–	9.8 μs^d^	[163]
**104** JM-6275	405^g^	525	PS	–		[165]
**105** JM-6280	283^g^	501	PS	–	3.1–12.2 μs^b^	[163]
**106** [Eu(tta)_3_phen]	268/341	612	PS-*co*-TFEM film	/	340–420 μs^h^	[12]
**107** [Eu(tta)_3_phencarz]	287/326	610	CH_2_Cl_2_	0.24–0.31^i^	0.15–0.53 ms^i^	[226]
**108** [Eu(tta)_3_DPIQ]	225/279	610	CH_2_Cl_2_	–	300 μs	[260]
**109** [Eu(tta)_3_ECIP]	260/350	610	PS film	–	380–440 μs^l^	[208]
**110** [Eu(DMB)_3_PIP]	256/293/353	610	CH_2_Cl_2_	0.18	215 μs	[138]
**111** [Eu(DMB)_3_IPD]	352	610	CH_2_Cl_2_	–	400 μs	[68]
**112** [Tb(acac)_3_(phen)]	228/268	546	Alumina film	–	–	[8]

^a^Measured in argon media and taken from [50]

^b^Absorption measured in CH_2_Cl_2_

^c^Fluorescence lifetime

^d^Under pure nitrogen

^e^In methanol

^f^In dichloromethane

^g^ *λ*_max_^exc^

^h^Range of lifetimes of the PS nanofibrous membranes doped with different amounts of complex **106** [253]

^i^Percentage of quantum yield and lifetimes of complex **107** in 1, 2, 3, and 4 wt% in PS in DMF solution

^l^Range of lifetimes of the nanofibrous membranes doped with different amounts of complex **109**

An analogous aluminum complex was shown to possess phosphorescence at room temperature once a heavy atom (iodine) was introduced in the ligand to promote intersystem crossing. The Al(III)-ferron complex **90** was either retained on the surface of anion exchange resin beads [144] or immobilized in sol–gel matrix to monitor oxygen in the gas phase, in water, and in organic solvents [50]; in both cases, the sensor was found to be very photo- and mechanically stable: no bleaching or leaching of the metal–chelate in either aqueous or organic solvent solutions was observed.

Strong room temperature phosphorescence under deoxygenation was also shown by boron difluoride dibenzoylmethane (BF_2_dbm) when coupled with poly(lactic acid) (PLA). This is rather surprising since virtually all boron complexes are known to be fluorescent but not phosphorescent (e.g., BODIPY dyes). In dichloromethane solution, BF_2_dbmPLA **91** showed a maximum absorption at 396 nm with a quite high molar absorption coefficient of about 50,000 M^−1^ cm^−1^ (Table 5), and an emission maximum at 436 nm with fluorescence quantum yield of 0.89 [256]. In solid state, on the other hand, BF_2_dbmPLA exhibits both delayed fluorescence and long-lived green room temperature phosphorescence, optical properties that can be exploited for single-component oxygen sensors.

In a more recent report, the same group modified the dually-emissive compound **91** to obtain the iodide-substituted difluoroboron dibezoylmethane-poly(lactic acid) compound **92** [258]. The addition of a heavy atom had the effect of enhancing the spin-orbit coupling and therefore increasing the rate of intersystem crossing, rendering **92** more practical for sensing applications. In fact, as shown in Table 5, the phosphorescence room temperature lifetime decreased from 170 to 4.06 ms. The sensitivity to oxygen of this boron biomaterials was demonstrated by fabricating both thin films and nanoparticles.

Recently, it has been shown that copper(I) complexes in crystalline form can be used as solid-state oxygen gas sensors and their sensing ability correlates well with the amount of void space contained within their lattice structures [205].

An advantage of these indicators is that they replace precious metals such as ruthenium, iridium, or platinum with copper which is significantly cheaper and oxygen-sensitive materials can be synthesized in few steps from commercially available starting material.

Copper complexes **93–95** (Fig. 15) are based on the same basic structure but differ by the substituent on the phenanthroline ligand (denoted with R) and by the other two ligands to the copper central atom (denoted with L).

[Cu(POP)(dmp)]tfbp **93** exhibits reasonable oxygen sensitivity but suffers from rapid photochemical degradation [205]; when the POP ligand (POP = *bis*[2-(diphenylphosphino)phenyl]ether) was substituted with the more rigid xantphos ligand (xantphos = 4,5-*bis*(diphenylphosphino)-9,9-dimethylxanthene), the stability of the resulting indicators increased. Complexes **93–95** possess good quantum yields (0.60–0.90) in the rigid matrices and lifetime in a range between 26–32 μs (Table 5).

Mann et al. [206] have recently shown that [Cu(CN-xylyl)_2_(dmp)] tfbp **96** and [Cu(TM4)(dmp)]_2_(tfbp)_2_ **97** can also be used as efficient crystalline oxygen sensors. These complexes showed much longer excited-stated lifetimes (in the order of ms) when compared to the previously reported [205] and therefore higher sensitivity to oxygen. It has been also reported that complex **97** shows higher stability under continuous illumination when compared to **96**.

Haitao et al. [88] recently constructed an optical sensor based on the sensitivity to oxygen of a novel Cu(I) complex: [Cu(PTO)(PPh_3_)_2_]BF_4_ **98**, which was embedded in mesoporous silica matrix. The photo-hysical properties of the complex are reported, in dichloromethane solution, in Table 5; the lifetime under pure nitrogen was found to be 64.4 μs which sharply decreases to 2.5 μs under 100 % O_2_ suggesting quite good sensitivity. When grafted onto silica matrix, the probe showed a red shift in the emission spectra from 515 to 554 nm due to a breakdown of the rigid structure of **98** when dispersed in the matrix; it was also demonstrated that the doping concentration has an influence on the sensitivity of the probe.

Very recently, a series of Cu(I) complexes based on indole derivatives (**99–102**; Table 5), were shown to possess dual emission—a feature that can be exploited for ratiometric fluorescence oxygen sensing [145].

All of these complexes, when in their solid state at room temperature did not show any emission because of triplet–triplet annihilation, but once dispersed in a rigid matrix like PMMA they showed quite good emission. The maximum emission peak of complexes **99**, **100**, **101**, and **102** are 562, 566, 608 and 610 nm, respectively, and can be assigned to the emission from the MLCT-excited states, which have a lifetime in the range of 1.10–1.50 μs. The second weaker emissions at lower wavelengths were found to have a much longer lifetime in the order of ms. Further developments of this new neutral Cu(I) complexes are currently under progress.

Generally, the main drawback of the Cu(I) complexes as oxygen indicators is their inefficient absorption in the visible part of the spectrum. In fact, most of the complexes can be efficiently excited only with the UV light.

Binuclear and polynuclear complexes of Au(I) were shown to exhibit strong visible luminescence when excited in the UV; this luminescence is associated with the Au–Au interaction which is thought to give rise to a metal-centered emission with excited state lifetime, in the absence of oxygen, between 4 and 200 μs [163]. Three different gold compounds are reported here: two containing two gold metal centers, the *bis*{μ-(*bis*(diphenylphosphino)octadecylamine-P,P′)} diiododigold(I) referred as JM6277 **103** and *bis*{μ-(*bis*(diphenylphosphino)octadecylamine-P,P′)} dichlorodigold(I) referred as JM6275 **104**, and one possessing a tripod structure containing only one gold metal center, the 1,1,1,-*tris*(2-diphenylphosphenitomethyl)-ethane gold(I) referred as JM6280 **105** (Fig. 16). Once incorporated in polystyrene, complex **103** showed an excitation maximum at 418 nm, an emission maximum at 530 nm, and an excited-state lifetime under nitrogen of 9.8 μs; the films showed good sensitivity to oxygen but their photostability was rather low since sunlight exposure caused complete photobleaching within 5 days [163]. When the iodine ligand of complex **103** was changed with chlorine, photobleaching was slightly reduced but still present especially after prolonged exposure to strong sunlight. Complex **104** was incorporated in both polystyrene (*λ*_max_^ex^ = 405 nm and *λ*_max_^em^ = 525 nm) and Ormosil matrices (*λ*_max_^ex^ = 360 nm and *λ*_max_^em^ = 525 nm) with latter showing a higher sensitivity to oxygen. The encapsulation in Ormosil resulted in a hypsochromic shift in the excitation maximum of 45 nm which suggests that the luminescence of the gold complex is affected by molecular interactions with the host matrix [165].

The mono gold(I) complex **105** showed strong luminescence not only in solid state but also in nonaqueous solvents like dichloromethane and tetrahydrofuran. Once incorporated in polystyrene, **105** showed an excitation maximum at 283 nm, an emission maximum at 501 nm and non-monoexponential decay curves with a short and a long lifetime components of 3.1and 12.2 μs, respectively.

Europium(III) complexes, thanks to their strong luminescence which is attributed to the 4*f*–4*f* transitions, have been widely applied in optical amplification, light conversion molecular devices, OLEDs, and their luminescence properties have been exploited for oxygen sensing [260]. Very narrow luminescence bands are of particular interest for oxygen sensing because of the potential for multiplexing applications where emission of other probes or labels can be used in different spectral windows.

A series of europium(III) complexes bearing similar structure have been recently reported and studied to be used as oxygen probes; complex **106–109** differ from each other by the diaza ligand as show in Fig. 15.

Amao et al. [12] immobilized [Eu(thenoyltrifluoroacetonato (tta))_3_phen] **106** in polystyrene-*co*-2,2,2-TFEM and characterized the resulting oxygen-sensitive film (Table 5). The [Eu(tta)_3_phen] films were shown to be sensitive to oxygen and possessed good photostability (no spectral changes after continuous irradiation using a 150 W tungsten lamp for 12 h). It has been reported that complex **106** suffers from photobleaching when used as powder but its photostability greatly increases once immobilized in rigid matrix [253]. Another group, in fact, used the same Eu(III) dye to dope a polystyrene nanofibrous membrane which resulted in a sensor for oxygen. Interestingly, the sensitivity increases with the weight percent of dopant up to 3 % [253].

Despite rather long luminescence lifetimes (several hundred microseconds), the sensitivity of the Eu(III) complexes is rather low. The luminescence of Eu(III) complexes is influenced by the energy gap between the Eu^3+^ ions and the antenna ligand and it is supposed to be more sensitive to the presence of oxygen when this energy gap is less than 1,500 cm^−1^ [129]. Therefore, a good way to increase the sensitivity of such complexes to oxygen is to change the nature of the ligand in order to modulate this energy gap.

 Recently the group of Li and Su [226] reported the preparation of an optical oxygen sensor based on [Eu(tta)_3_phencarz] **107**, which has a metal-ligand energy gap of 721 cm^−1^. The complex was electrospun in polymer nanofibrous membrane. In this case as well, the sensitivity of the sensor showed a dependence on the amount of dye (weight percent) used to dope the nanofibrous membrane (lifetimes were in order of ms) and the best performances were obtained with 3 % of **107** in the matrix.

Two further complexes featuring three tta ligands and an additional antenna ligand have been reported: [Eu(tta)_3_DPIQ] **108** and [Eu(tta)_3_ECIP] **109** which were respectively incorporated in a mesoporous matrix and in polystyrene nanofibrous membrane [208, 260]. The sensitivity to oxygen of these two complexes was lower than the one of complex **107**; the photophysical properties can be found in Table 5.

Complexes **110** and **111** (Fig. 16) possess the same basic structure (the tta ligands are changed with DMB (1,3-diphenyl-propane-1,3-dione)) but differ from each other by the diaza ligand. [Eu(DMB)_3_PIP] **110** was electrospun into poly(vinypyrrolidone) [138] while [Eu(DMB)_3_IPD] **111** was used to dope silica matrix [68]. Both were shown to have good sensitivity toward oxygen and to be potentially useful, once incorporated into the matrix, as optical oxygen sensors.

The strong luminescence of another lanthanide complex has been exploited by Amao et al. [8] to fabricate optical oxygen sensing material: the *tris*-(acethylacetonato)(1,10-phenanthroline) terbium(III) complex **112**.

The luminescence lifetime of Tb(III) complexes are usually quite long (up to several ms) and the quantum yields often about 50 %, but the actual values for **112** were not reported by the authors. The major drawback of this indicator is that it cannot be excited in the visible light but only in the UV region because the main resonance level of terbium ^5^D_4_ is located at rather high energies (~20,400 cm^−1^).

It should be mentioned here that for the similar reasons, the excitation of most Eu(III) complexes is also located in the UV region which is a serious drawback. Additionally, it should be noted that the luminescence of the Eu(III) complexes is usually strongly affected by temperature, which enabled their application as optical thermometers and their use for simultaneous luminescent sensing of temperature and oxygen [29].

Very recently, an oxygen indicator based on a gadolinium(III) complex was reported [26]. The acridone antenna was covalently bound to a polystyrene backbone to enable excitation with violet light. Gadolinium(III) *tris*-thenoyltrifluoroacetonate complex was used as an energy acceptor and showed rather strong phosphorescence (*Φ* = 14 %) at room temperature in the absence of oxygen which is attributed to efficient intersystem crossing due to the heavy atom effect and paramagnetism of Gd(III). Quenching by oxygen was rather efficient due to a long decay time of about 900 μs, but Stern–Volmer plots showed pronounced nonlinearity indicating high heterogeneity of the material. Nevertheless, the work demonstrated for the first time the potential of Gd(III) complexes as new phosphorescent indicators for oxygen sensors.

6. Miscellaneous indicators

In this section, the reader will find all those indicators that do not belong to any specific class but still have been successfully used for optical oxygen sensing.

The thermally activated E-type delayed fluorescence (DF) of fullerene C_70_ **113** (Table 6) has been exploited by Nagl et al. [169] to make an optical oxygen sensor that is especially suited for sensing oxygen down to the ppb range. High sensitivities are explained by exceptionally long decay time of the delayed fluorescence (20 ms). The DF quantum yield of **113** increases from 0.01 at 20 °C to 0.08 at temperatures around 150 °C. Fullerene C_70_ was incorporated into highly oxygen-permeable matrices such as organically modified silica and ethyl cellulose to enable good sensitivity. Another feature is high chemical and photochemical stability of the material; on the other hand, moderate luminescence brightness (particularly at room temperatures) is a clear disadvantage.

**Table 6:** Photophysical properties of miscellaneous indicators

Dye	*λ*_max_^abs^ (nm) (*ε* × 10^3^ M^−^ cm^−1^)	*λ*_max_^em^ (nm)	Medium	*Φ*	*τ*	Reference
**113** Fullerene C_70_	470 (20)	650–725	Organosilica	0.01	20 ms	[169]
**114** [Mo_6_Cl_8_]Cl_4_L_2_	300–400	600–900	PTMSP^a^	–	100 μs	[82]

^a^Poly[1-trymethylsilyl-1-propyne]

The photophysical properties of molybdenum cluster **114** (Table 6) were shown to be well suited for oxygen sensing, as the red luminescence of the Mo cluster in PTMSP (poly[1-trymethylsilyl-1-propyne]) can be efficiently quenched by oxygen. The luminescence intensity can be easily detected by integration over the broad emission band as the cluster exhibits quite a long lifetime (>100 μs) and a large Stokes’ shift (300 nm) [82]. The advantage of Mo cluster is that they and show exceptional thermal stability with no sign of decomposition even at temperatures higher than 600 °C.

Mo clusters were modified to be used for aqueous applications: K_2_Mo_6_Cl_14_ clusters were caged in a hydrophobic oxygen polymer matrix, the [(acryloxypropyl)-methylsiloxane-dimethylsiloxane copolymer] [81]. A fiber optic sensor based on the phosphorescence quenching of K_2_Mo_6_Cl_14_ clusters has been developed and if showed no photobleaching after more than 13,000 measurements and it gave a linear response in the temperature range between 10 and 37 °C.

The dependence of the fluorescent spectra of poly(9,9-dioctylfluorene) (PF8) on the oxygen content has been recently exploited to create an optical oxygen gas sensor [15]. It has been shown that in addition to the irreversible oxidation of PF8, which results in the formation of Keto defects, reversible fluorescence quenching is also observed when the PF8 thin films are excited with a He–Cd laser at 325 nm. The sensitivity of the conjugated polymer was only moderated and unfortunately, the sensors based on PF8 degrade irreversibly after 12 h of continuous laser exposure.

## Criteria for selection of indicators

As it has been highlighted, numerous classes of oxygen indicators exist and feature significantly different photophysical and sensing properties. After giving an overview of the available indicators for optical oxygen sensing, in order to help the reader to choose the most suitable one for his/her needs, useful criteria for selection will be given in this session.

### Absorption and emission spectra

In general, excitation in the UV part of the spectrum should be avoided due to high levels of background fluorescence originating from many biological substances, sensor supports, optical components etc. Additionally, UV light can be often disturbing for many biological systems. These are among the reasons why indicators excitable in the visible part of the spectrum are strongly preferable. Oxygen monitoring in tissues is more demanding and requires indicators that can be excited in the red or NIR part of the spectrum; this ensures deeper penetration depth of the excitation light and minimizes the loss of the emission light. As was demonstrated above, Pt(II) and Pd(II) complexes with π-extended benzo-naphthoporphyrins as well as azabenzoporphyrins represent an excellent choice for such applications.

Generally, large Stokes’ shifts are strongly preferable since the emission can be easily separated from the excitation light by means of optical filters. Fortunately, this requirement is almost always fulfilled for luminescent indicators. Compatibility with the available excitation sources and detectors is another important issue. Depending on the application the excitation sources of choice can be LEDs, laser diodes, lasers or, e.g., a mercury lamp. For example, bright LEDs are now available for virtually all parts of the spectrum; however, there is a gap between 540 and 580 nm. Compatibility with the detector can be another critical issue. For example, oxygen imaging with NIR-emitting dyes can be critical because of the lower sensitivity of the CCD chips in this region. The same is even more crucial for the photomultipliers as most devices (e.g. confocal microscopes) are still equipped with cheaper models which become almost insensitive at λ>700 nm. Finally, compatibility with other optical components can be important for some particular sensors. For instance, the attenuation of cheap plastic optical fibers peaks at about 740 nm and then further increases above 820 nm, which can represent a certain problem when using NIR dyes. Fortunately, benzoporphyrin complexes emit between 770 and 800 nm, where the attenuation is lower.

### Luminescence brightness of the probe

The luminescent brightness is defined as the product of the molar absorption coefficient (ε) and the luminescence quantum yields (*Φ*). Therefore, an indicator possessing a quantum yield close to unity but a molar absorption coefficient of only several thousand moles per centimeter can still not be bright enough for some applications. This situation is typical for most cyclometallated complexes of Ir(III) and Pt(II).

Generally, an advantage of indicators with high luminescence brightness is that they can be used in lower concentration within the polymeric matrix (thus avoiding aggregation) and therefore enable the preparation of fast responding thin-film planar optodes or optical fibers. Nanoparticle sensors based on bright indicators can be used in lower concentration thus providing less interference to the biological systems. High luminescence brightness is of particular importance for measurements in tissues since the loss of excitation and the emission light due to absorption and scattering is very significant.

Clearly, the oxygen indicators with the highest brightness reported are the orange light-emitting Ir(III) coumarin complexes and NIR emitting Pt(II) benzoporphyrins. In fact, these dyes possess both very high molar absorption coefficients (>80,000 M^−1^ cm^−1^) and luminescence quantum yields (>0.5). The brightness of the not extended Pt(II) porphyrins (e.g., octaethylporphyrin) is also very high but only upon excitation in the Soret band located in the UV region.

### Luminescence decay times

The luminescence decay time of an indicator is also a very important parameter since the sensitivity of an optical oxygen sensor is proportional to both the decay time of the indicator and the gas permeability of the polymer. Thus, indicators with moderately long decay times (several microseconds) will show sufficient sensitivity only in highly gas-permeable matrices (silicone rubber, fluorinated polymers, or Ormosils) but in many common polymers used for the preparation of planar optodes, fiber-optic sensors, and nanoparticles (e.g., polystyrene) they will not show adequate sensitivity. On the other hand, long decay times (>1 ms) are often too long to design sensors which can operate between 0 and 21 kPa pO_2_. In fact, the luminescence of such indicators will be almost completely quenched at low pO_2_; in these cases, a solution could be to use polymers with low gas permeability even though it should be considered that the dynamic response of such materials will be negatively affected. Conversely, indicators with long decay times are indispensible when designing trace oxygen sensors. In fact, the sensors for moderately low oxygen concentrations can be designed on the basis of Pd(II) porphyrins (decay times about 1 ms) but ultratrace oxygen sensors require indicators with even longer decay times. Fullerene C70 (*τ* = 20 ms) was found to be useful for such applications. On the other hand, Pt(II) porphyrins with their phosphorescence decay times of 40–80 μs represent most popular indicators for designing oxygen sensors operating in physiologically relevant concentrations.

### Chemical stability and photostability

In general, the chemical stability of the majority of the indicators used for oxygen sensing is acceptably good. However, their stability in harsh conditions (high temperature and humidity during sterilization, presence of oxidizing species such as hypochlorite, etc.) can be poor.

On the other hand, the photostability of different dyes can vary a lot even among compounds that are chemically closely related. This property is not critical for indicators used in disposable systems but it is fundamental for those applications where high light intensities are used and/or prolonged measurements are performed. Photosensitized singlet oxygen is often responsible for the photodegradation of indicator dyes through oxidation; therefore the introduction of electron-withdrawing substituents (such as halogens) in the indicator molecule usually helps to improve their photostability. For this reason, the complexes of Pt(II) and Pd(II) with highly fluorinated tetra(pentafluorophenyl)porphyrin are known to be one of the most photostable indicators reported. Unfortunately, very little data comparing the photostabilities of different classes of oxygen indicators is available.

### Cross-sensitivity to other parameters

The luminescence of an indicator can be affected by the presence of ionic species and water. This can be critical for water-soluble indicators but it is of less relevance for the lipophilic ones since they are incorporated in polymers which can act as permeation-selective membrane for ionic species.

The phosphorescence of all luminescent dyes is prone to thermal quenching; however, the extent of this process varies dramatically for different indicator groups. Particularly, MLCT indicators (e.g., Ru(II) polypyridyl complexes) and luminescent Eu(III) complexes show a much higher degree of thermal quenching than, for example, Pt(II) metalloporphyrins (0.05–0.2 % of the decay time change per 1 K). The temperature cross-sensitivity of oxygen-sensing materials does not only depend on the nature of the compound but mostly is dominated by the temperature dependence of the diffusion and solubility of oxygen in polymers.

### Solubility in the polymeric matrices/analyzed media

Low solubility of an indicator in the polymeric matrix can result in either aggregation or migration to other components (e.g., the material of the sensor support). Aggregation is of particular concern in case of planar π-extended molecules such as, e.g., unsubstituted porphyne, tetrabenzoporphyrin or phthalocyanine. The solubility in apolar polymers can usually be enhanced by introducing bulky substituents in the indicator molecule (e.g., alkyl chains). In case of porphyrins, it is efficiently suppressed by introducing phenyl rings in the *meso*-position of the porphyrin macrocycle. On the contrary, polar groups (e.g., charged groups, polyethylene glycol chains, peptides or proteins, etc.) can be introduced to enable/enhance the solubility of an indicator in water. An alternative method to suppress dye migration and leaching is to covalently grafting the indicator into the matrix. However, often tedious modification of the indicator is necessary to achieve this goal. It should be mentioned here that the solubility in the analyzed media can also be obtained by immobilizing lipophilic indicators in the core of amphiphilic polymeric nanoparticles bearing polar groups on their surface. In this case modification of an indicator is not necessary.

### Toxicity

Toxicity of an indicator is of main concern for biological and medical applications. Usually, encapsulation of the indicator into a polymeric matrix greatly reduces its toxicity. However, the phototoxicity caused by the production of singlet oxygen cannot be eliminated completely. This is especially true for water-soluble indicators and nanoparticles since the diffusion ways are short enough for the singlet oxygen to damage the cells despite its relatively short lifetime (about 3 μs) in aqueous media. Here, indicators with exceptional brightness are very useful since the loading of the probe (and consequently damage for the live cells) can be substantially reduced without compromising the S/N ratio.

### Commercial or synthetic availability

Commercial availability is very important for numerous researchers who do not have enough facilities/experience to prepare the oxygen indicators by themselves. Unfortunately, only few oxygen indicators are commercially available at acceptable prices, such as, for example, the Ru(III) polypyridyl complexes and Pt(II) and Pd(II) complexes with some porphyrins including OEP and TFPP. If the synthesis of the indicators cannot be avoided the simplicity of the method is often of crucial significance. Price and/or simplicity of the synthetic pathway are of much less importance if the indicators are used in microscopic imaging or in fiber-optic microsensors because the amount of dye needed is usually very small. On the other hand, some emerging applications (e.g., food packaging) would require high quantities of the indicators at very competitive price. In this case, the less conventional indicators such as copper(I) or lanthanide(III) complexes can be very promising providing that they possess efficient absorption in the visible or NIR part of the spectrum and acceptable luminescence quantum yields.

## Concluding remarks

To summarize, there is no perfect indicator which would be suitable for all oxygen-sensing applications. An adequate indicator should rather be carefully chosen according to the above mentioned criteria. Among different dye classes, Pt(II) and Pd(II) complexes with benzoporphyrins can be considered the most promising due to the high flexibility in tuning their spectral properties but also because of excellent luminescent brightness and photo-stability of some representatives.

These dyes are also suitable for further synthetic modification to provide additional functionalities. However, certain representatives of other classes possess interesting photophysical and sensing properties, such as for example ultrabright emission (iridium(III) coumarin complexes), dual emission (some platinum(II) complexes) which makes them particularly promising for ratiometric imaging. Despite the significant progress in the field of oxygen indicators that has been achieved in the last decade, there is still work to be done particularly in designing tailor-made dyes for many vital applications where state-of-the-art systems may not be fully adequate.
